# Therapeutic targeting of local metabolic dysfunction in secondary lymphedema: mechanisms and opportunities

**DOI:** 10.3389/fphys.2026.1865368

**Published:** 2026-07-21

**Authors:** Kechen Ye, Lingli Jiang, Junzhe Chen, Shaoxiang Yuan, Tong Xiao, Yuezhong Chen, Tao Xiong, Ziyi Luo, Chengliang Deng, Shun’e Xiao

**Affiliations:** Department of Burns and Plastic Surgery, Affiliated Hospital of Zunyi Medical University, Zunyi, China

**Keywords:** energy metabolism, lipid metabolism, lymphatic system, metabolic dysfunction, secondary lymphedema, therapeutic targets

## Abstract

Secondary lymphedema is a chronic and progressive disorder resulting from impaired lymphatic drainage after surgery, radiotherapy, infection, trauma, or other lymphatic insults. Its pathological features include chronic inflammation, adipose deposition, fibrosis, and structural and functional damage to lymphatic vessels. Previous studies have mainly explained disease progression from the perspectives of lymphatic injury and tissue remodeling, while local metabolic dysfunction and the associated immune alterations remain insufficiently defined. Recent evidence indicates that lymphedematous tissues exhibit disturbances in both energy metabolism and lipid metabolism. These metabolic changes can impair the survival, proliferation, and reparative capacity of lymphatic endothelial cells and may also promote inflammation, adipose deposition, and fibrosis by regulating immune-cell behavior and function. This review systematically summarizes recent advances in local metabolic dysregulation in lymphedema, discusses pharmacological strategies targeting metabolic pathways and their current translational limitations, and aims to provide a conceptual framework for future studies on immunometabolic mechanisms and therapeutic interventions in secondary lymphedema.

## 1 Introduction

Lymphedema is a chronic progressive disease characterized clinically by persistent limb swelling and impaired mobility, with progressive effects on appearance, psychological well-being, and quality of life. Epidemiologic data suggest that lymphedema affects hundreds of millions of individuals worldwide (Kim et al., 2024). According to etiology, lymphedema can be classified as primary or secondary. Primary lymphedema is associated with inherited abnormalities of lymphatic development and is mainly manifested by lymphatic hypoplasia or functional defects (Martin-Almedina et al., 2021). Secondary lymphedema results from structural or functional injury to lymphatic vessels or lymph nodes caused by surgery, radiotherapy, infection, trauma, or related factors, leading to impaired lymphatic drainage (Murdaca et al., 2012). Current clinical management relies on combined surgical and conservative approaches. Surgical procedures include lymphaticovenous anastomosis, vascularized lymph node transfer, and liposuction-based debulking, whereas conservative treatment mainly includes compression therapy and manual lymphatic drainage. These interventions can reduce swelling to some extent, but no widely accepted curative treatment is currently available.

Most current studies have focused on lymphatic injury, inflammation, lipid deposition, and fibrosis, while the mechanisms underlying local metabolic abnormalities remain insufficiently explored. Local metabolic dysregulation in lymphedema appears to be stage dependent. In the early phase, lymphatic injury causes interstitial fluid retention, which then induces hypoxia, inflammatory infiltration, and local metabolic reprogramming, typically as a secondary adaptive response. As the disease progresses, these alterations may evolve into persistent metabolic imbalance, further impair lymphatic structure and function, and drive disease progression (Norden and Kume, 2020). It is therefore necessary to clarify the pathogenesis of lymphedema from the perspective of reciprocal interactions between lymphatic injury and local metabolic imbalance. This review focuses on the relationship among lymphatic injury, tissue remodeling, and metabolic abnormalities in lymphedema, systematically summarizes major mechanisms of energy and lipid metabolic dysfunction, and discusses related metabolic interventions to provide a theoretical basis for targeted treatment.

## 2 Review methodology and search strategy

A structured literature search was conducted using the PubMed database. The search included studies published from database inception to April 2026 and focused on original research and relevant reviews concerning secondary lymphedema, lymphatic dysfunction, metabolic dysregulation, immunometabolism, inflammation, fibrosis, adipose remodeling, and metabolism-oriented therapeutic strategies.

The main search terms included “lymphedema,” “lymphoedema,” “lymphatic endothelial cell,” “lymphangiogenesis,” “immunometabolism,” “metabolic dysfunction,” “hypoxia,” “glycolysis,” “mitochondrial dysfunction,” “oxidative stress,” “fatty acid oxidation,” “ketone body oxidation,” “amino acid metabolism,” “cholesterol,” “lipid mediator,” “adipokine,” “macrophage polarization,” “T cell,” “Treg,” “fibrosis,” “adipose deposition,” “metformin,” “PPARγ agonist,” “GLP-1 receptor agonist,” “ketoprofen,” “tacrolimus,” “selenium,” “ketogenic diet,” and “exercise.”

Human studies of secondary lymphedema were prioritized when available, including clinical studies, observational cohorts, case reports, and interventional trials. Animal models and *in vitro* studies were included when they provided mechanistic insight into lymphatic endothelial cells, immune regulation, inflammatory remodeling, or metabolic pathways relevant to lymphedema. Evidence extrapolated from obesity, diabetes, cardiovascular disease, or general inflammatory disorders was considered only when direct lymphedema-specific evidence was limited and was interpreted cautiously. In addition to the database search, reference lists of relevant articles were manually screened to identify additional eligible studies. Publications were included if they were written in English and were directly relevant to lymphatic function, local metabolism, immune regulation, inflammation, fibrosis, adipose remodeling, or therapeutic targeting in lymphedema.

## 3 Development of the lymphatic vessel and maintenance of tissue homeostasis

Lymphatic vessel development begins during embryogenesis when a subset of venous endothelial cells acquires a lymphatic endothelial phenotype (Wigle et al., 2002; Oliver and Srinivasan, 2010). PROX1 is a core transcription factor required for lymphatic endothelial cell differentiation and phenotypic maintenance (Wigle and Oliver, 1999), and its expression and activity are regulated mainly by SOX18 and COUP-TFII (Srinivasan et al., 2010; Yang et al., 2012). Sprouting, migration, and proliferation of lymphatic endothelial cells depend largely on the VEGF-C/VEGFR3 signaling axis (Hong et al., 2002; Srinivasan et al., 2014). VEGF-C is a key growth factor for early lymphatic sprouting and lymphatic network formation (Karkkainen et al., 2004). VEGFR3, the major receptor for VEGF-C, is highly expressed in lymphatic endothelial cells and regulates their survival, proliferation, migration, and lumen formation (Kukk et al., 1996). After the initial lymphatic vessels form, the lymphatic network undergoes structural remodeling and functional maturation, ultimately differentiating into initial and collecting lymphatic vessels. Initial lymphatic vessels are composed of oak leaf-shaped lymphatic endothelial cells with overlapping borders and discontinuous button-like junctions, which facilitate the entry of interstitial fluid, proteins, and immune cells. Collecting lymphatic vessels rely on smooth muscle contraction and valves to drive unidirectional lymph flow (Baluk et al., 2007; Baluk and McDonald, 2022).

Mature lymphatic vessels play essential roles in fluid balance, immune regulation, and metabolic homeostasis (Oliver et al., 2020). To maintain fluid balance, fluid, proteins, and certain metabolites filtered into the interstitium must return to the blood circulation through the lymphatic system (Aukland and Reed, 1993). In immune regulation, lymphatic vessels mediate antigen transport, immune-cell trafficking, and the organization of adaptive immune responses within lymph nodes. Dendritic cells and T cells in peripheral tissues can migrate through lymphatic vessels to draining lymph nodes (Randolph et al., 2017). Lymphatic vessels also participate in multiple metabolic processes, particularly lipid absorption, lipid transport, and peripheral cholesterol return. Intestinal lacteals absorb long-chain fatty acids and transport them into the circulation as chylomicrons (Cifarelli and Eichmann, 2019; Wilting and Becker, 2022), while cholesterol and related lipids in peripheral interstitial spaces require lymphatic drainage for reverse transport (Lim et al., 2026). Thus, the lymphatic vasculature is a key component of peripheral lipid homeostasis.

When lymphatic structure is damaged or lymphatic function declines, interstitial retention of fluid, proteins, and lipid-related components can lead to chronic inflammation, extracellular matrix deposition, and expansion of adipose tissue (Lim et al., 2026). Recent studies further indicate a close pathological connection between lymphatic dysfunction and metabolic dysfunction: lymphatic dysfunction aggravates local metabolic dysregulation, and local metabolic abnormalities can in turn erode lymphatic structure and function (Jiang et al., 2019). The lymphatic vasculature is therefore a structural basis for interstitial fluid return and a critical component in maintaining local metabolic homeostasis and tissue microenvironmental balance.

## 4 Pathological basis of tissue remodeling in lymphedema

Impaired lymphatic drainage causes retention of protein- and lipid-rich interstitial fluid, disrupting local microenvironmental homeostasis and producing pathological changes characterized by inflammation, fibrosis, and lipid deposition. Inflammation is an important driver of tissue remodeling during both early and progressive disease. CD4+ T cells are considered a key cell population driving chronic inflammation and tissue remodeling. Studies show that CD4+ T cells can be activated in regional draining lymph nodes, migrate to the skin and affected tissues, and display enhanced Th1/Th2-related differentiation and increased expression of chemokines and adhesion molecules; depletion of CD4+ T cells in mouse models reduces local tissue inflammation in lymphedema (Zampell et al., 2012b; García Nores et al., 2018). Treg-mediated immune regulation also helps limit local inflammatory responses (Gousopoulos et al., 2016). Abnormal T-cell activation and subset imbalance therefore contribute to the establishment and persistence of the inflammatory microenvironment in lymphedema. In addition to CD4+ T cells, macrophages are important inflammatory cells in lymphedematous tissues. They can accumulate around damaged adipocytes and release inflammatory cytokines such as TNF-alpha and IL-6 (Tashiro et al., 2017), while also contributing to lymphatic repair and anti-fibrotic responses (Ghanta et al., 2015). Chronic inflammation mediated by these immune cells provides a microenvironmental basis for subsequent fibrosis and lipid deposition.

Under sustained inflammatory infiltration and lymph stasis, local tissues begin to undergo fibrotic remodeling. Thickening of fibrous septa between adipose lobules and collagen deposition have been observed in both patient tissues and animal models, indicating that fibrosis and extracellular matrix remodeling are important histological features of lymphedema (Zampell et al., 2012b; Gardenier et al., 2016). Among the profibrotic regulators identified, TGF-beta1 has been extensively studied. Its upregulation promotes ECM deposition and dermal fibrosis, thereby driving the transition from early, potentially reversible edema to chronic irreversible disease (Baik et al., 2022; Park et al., 2025).

In addition to inflammation and fibrosis, adipose deposition is another core feature of tissue remodeling in lymphedema. Multiple studies suggest that persistent lymphatic drainage failure gradually leads to adipose tissue thickening, adipocyte enlargement, and cholesterol accumulation in affected tissues (Brorson et al., 2006; Zhang et al., 2022; Mehrara, 2026). The mechanism of lipid deposition is generally considered to involve hypertrophy of mature adipocytes and abnormal lipid retention, although whether abnormal proliferation of adipose precursor cells also occurs remains debated. Early studies reported that adipose-derived stem cells from lymphedema tissues possess enhanced adipogenic differentiation potential (Levi et al., 2013; Xiang et al., 2020; Hsiao et al., 2023). More recent single-cell RNA sequencing studies, however, did not detect marked upregulation of adipogenic genes or pathways in lymphedema adipose-derived stem cells, indicating that the underlying mechanisms require further investigation (Liu et al., 2022). Taken together, inflammatory immune infiltration, fibrosis and extracellular matrix remodeling, and adipose deposition are interwoven pathological foundations of tissue remodeling in lymphedema and provide the histological background for discussing local metabolic dysfunction.

## 5 Mechanisms of metabolic dysfunction in lymphedema

**Figure 1:**
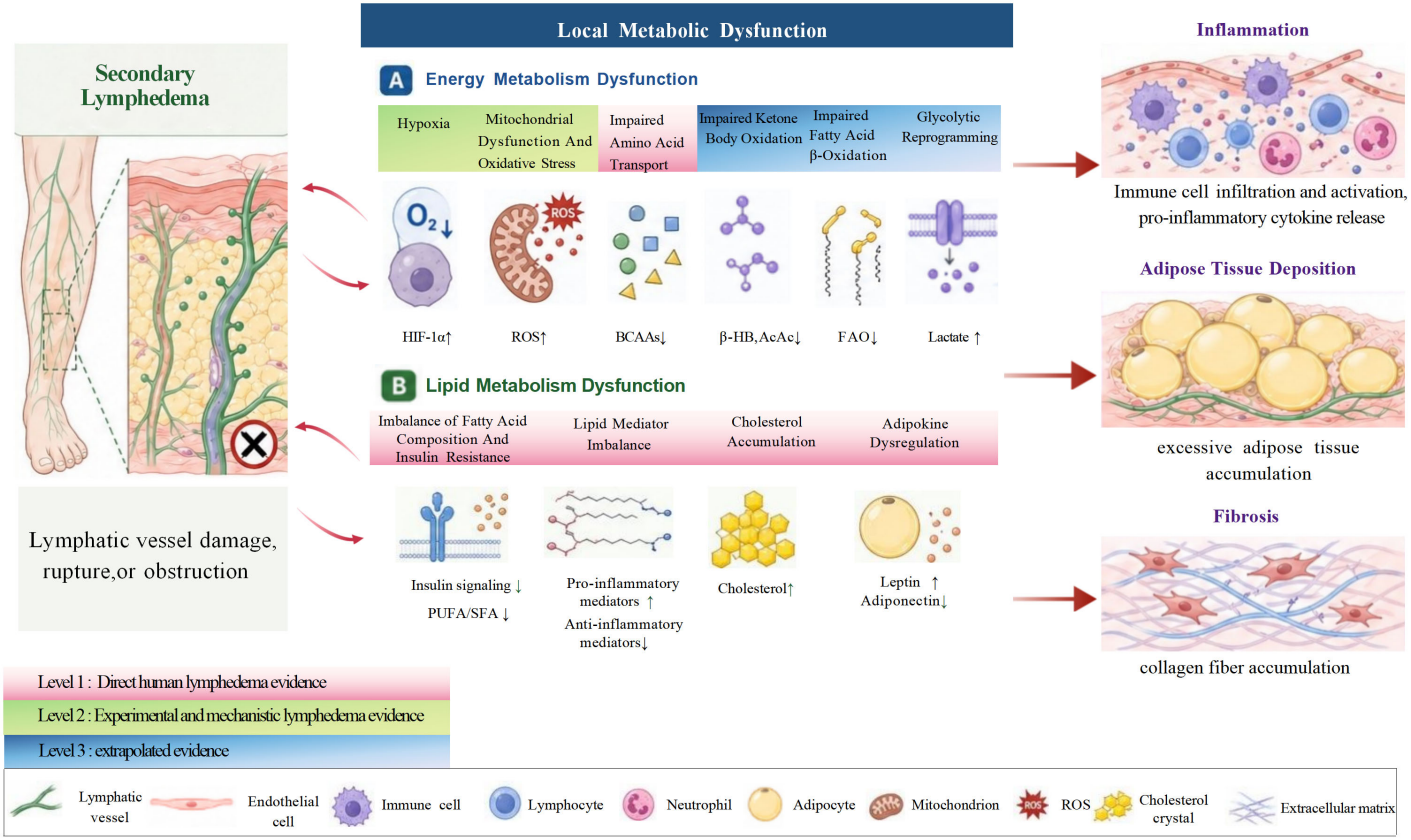
Interplay between lymphatic dysfunction and metabolic reprogramming in secondary lymphedema.

On the basis of persistent lymphatic drainage failure, the local tissue microenvironment gradually develops a metabolic state characterized by energy metabolic dysfunction and lipid metabolic dysregulation, which continuously participates in the course of lymphedema progression (**Figure 1**).

### 5.1 Energy metabolism dysregulation

#### 5.1.1 Hypoxia

Long-term lymph accumulation can increase interstitial pressure, impair local microcirculation, and promote inflammatory-cell infiltration, thereby generating a hypoxic microenvironment. In the context of lymphedema-related energy metabolism, hypoxia is both a hallmark of local microenvironmental imbalance and an upstream trigger of metabolic adaptation. Hypoxia-inducible factors (HIFs) are heterodimers composed of an alpha subunit (HIF-1alpha or HIF-2alpha) and a beta subunit (Lee et al., 2020; Jiang et al., 2022). Under hypoxic conditions, HIF-1alpha activation promotes glucose uptake and glycolytic pathways while inhibiting pyruvate entry into the tricarboxylic acid cycle, shifting cellular energy production from mitochondrial oxidative phosphorylation toward glycolysis to meet basic energy needs under low oxygen tension.

Hypoxia has stage-dependent effects on lymphatic endothelial cells (LECs). In the early disease phase, moderate hypoxia may induce VEGF-C/VEGFR3 expression through HIF-1alpha and thereby promote reparative lymphangiogenesis (Min et al., 2011; Zampell et al., 2012a). When local hypoxia persists, sustained HIF-1alpha elevation together with relative insufficiency of HIF-2alpha may lead to nonfunctional lymphangiogenesis, which fails to improve drainage and may aggravate tissue edema. Beyond its effects on LECs, hypoxia can also promote adipose inflammation and immune imbalance. Jun et al. showed that hypoxia induces adipocyte death and macrophage recruitment (Yin et al., 2009), and these infiltrating cells are mainly regulated by HIF-1alpha, mediating insulin resistance and inflammation (Mazzatti et al., 2012). In hypoxic lymphedematous tissues, adipose tissue displays an inflammatory phenotype similar to that seen in obesity, characterized by increased TNF-alpha, IL-6, and IL-1beta. By contrast, HIF-2alpha enhances Vegfa and Irs2 expression, helping maintain adipose tissue angiogenesis and insulin sensitivity (García-Martín et al., 2016). HIF-1alpha also promotes M1-like macrophage polarization and Th17 differentiation and suppresses the key Treg gene FOXP3, whereas HIF-2alpha helps maintain M2-like macrophages and Treg stability (Imtiyaz et al., 2010; Dang et al., 2011; Fujisaka et al., 2013; Hsu et al., 2020).

Overall, hypoxia can induce compensatory metabolic adaptation during the early phase of disease to support cell survival. Persistent hypoxia, however, enhances glycolysis and impairs mitochondrial function, gradually converting early metabolic adaptation into energy metabolic imbalance and thereby promoting disease progression.

#### 5.1.2 Glycolytic reprogramming

The energy supply of lymphatic endothelial cells is primarily glycolytic. This metabolic pattern rapidly generates ATP and provides biosynthetic intermediates required for cell proliferation, migration, and lymphatic sprouting (Wong et al., 2017; Yu et al., 2018; Jiang et al., 2021). FGFR signaling is an important upstream regulator of this phenotype (Yu et al., 2017). Inhibition of FGFR1 reduces glycolysis and ATP production in LECs while inducing a compensatory increase in fatty acid beta-oxidation (FAO), indicating that glycolysis is the dominant energy pathway in LECs and that fatty acid oxidation may act as a compensatory pathway when glycolysis is suppressed (Wong et al., 2017).

Maintenance of glycolysis requires coordinated regulation by multiple key enzymes. Among glycolytic enzymes, hexokinase 2 (HK2) and pyruvate kinase M2 (PKM2) are critical for preserving lymphatic endothelial structure and function. HK2 is an important rate-limiting enzyme at the beginning of glycolysis and converts glucose into glucose-6-phosphate. Simeroth et al. showed that loss of HK2 in mice impairs lymphatic development (Simeroth and Yu, 2024). PKM2 is also indispensable for LEC function. Jiang et al. reported that PKM2 deletion in LECs suppresses lymphatic endothelial structure and function, and pharmacological inhibition of PKM2 reduces lymphatic malformations in a rat model (Jiang et al., 2021). Thus, glycolysis is a major pathway for basal energy supply in LECs and a key metabolic foundation for normal lymphatic development and functional homeostasis.

In the hypoxic microenvironment of lymphedema, glycolytic reprogramming may increase lactate production while reducing mitochondrial oxidation of pyruvate, thereby altering the local metabolic milieu (Jiang et al., 2021). High concentrations of lactate can promote fibroblast-to-myofibroblast differentiation and extracellular matrix deposition through TGF-beta signaling (Xie et al., 2015), and lactate can also serve as a substrate for histone lactylation and epigenetic regulation (Zhang et al., 2019). Glycolytic reprogramming therefore affects the energy supply of lymphatic endothelial cells and may contribute to local fibrosis through lactate accumulation.

#### 5.1.3 Mitochondrial dysfunction and oxidative stress

Under sustained hypoxia and chronic inflammatory stimulation, local cellular metabolism in lymphedema shifts from oxidative phosphorylation toward glycolysis and is accompanied by mitochondrial dysfunction. Mitochondria are major sites of oxidative phosphorylation and reactive oxygen species generation and are essential for maintaining the energy supply of lymphatic endothelial cells. Ma et al. showed that mice lacking QPC, a key subunit of mitochondrial respiratory chain complex III, exhibit reduced expression of the lymphangiogenic gene VEGFR3 (Ma et al., 2021), leading to impaired lymphatic structure and function. This effect was associated with reduced H3K4me3 and H3K27ac levels at the Vegfr3 and Prox1 loci, suggesting that mitochondrial dysfunction can impair LEC structure and function through both energetic and epigenetic mechanisms.

On the basis of mitochondrial dysfunction, local redox homeostasis may become further disrupted, aggravating cellular injury and inflammation and thereby contributing to lymphedema progression (Turrens, 1997; Chang et al., 2013). Clinical studies have shown decreased reduced glutathione (GSH), increased oxidized glutathione (GSSG), and marked elevation of lipid peroxidation products including malondialdehyde (MDA) and 4-hydroxynonenal (HNE) in patients with lymphedema (Siems, 2002). After lymphaticovenous anastomosis improves lymphatic drainage, expression of oxidative stress-related genes decreases (Yang et al., 2021). Oxidative stress also impairs the reparative capacity of LECs. Hossain et al. found that in an oxidative stress microenvironment, VEGF-C fails to promote LEC proliferation and instead activates p53 through ROS generation and DNA damage, resulting in LEC apoptosis (Hossain et al., 2024).

Thus, mitochondrial dysfunction and oxidative stress can form a mutually reinforcing vicious cycle in the metabolic microenvironment of lymphedema. Oxidative stress initiated by mitochondrial dysfunction promotes loss of mitochondrial membrane potential, DNA damage, and apoptosis, while oxidative stress further damages mitochondrial function and inhibits lymphangiogenesis (Sanz et al., 2006; Ježek et al., 2018). Mitochondrial dysfunction and oxidative stress impair LEC energy metabolism and obstruct lymphatic regeneration through inflammatory injury and apoptosis, jointly promoting disease progression.

#### 5.1.4 Impaired fatty acid β-oxidation

Fatty acid beta-oxidation (FAO) is one of the core pathways of lymphatic endothelial energy metabolism and is important for phenotypic maintenance and injury repair. FAO generates acetyl-CoA to fuel the tricarboxylic acid cycle (TCA) and provides precursors such as glutamate and aspartate for *de novo* synthesis of purine and pyrimidine deoxynucleotides (dNTPs) (Schoors et al., 2015).

Existing evidence indicates that FAO has a key regulatory role in lymphatic development. Wong et al. found that LEC-specific deletion of carnitine palmitoyltransferase 1A (CPT1A), the rate-limiting enzyme of FAO, impairs lymphangiogenesis (Wong et al., 2018). During early LEC differentiation, COUP-TFII activates and maintains Prox1 expression (Srinivasan et al., 2010), while PROX1 upregulates CPT1A to enhance FAO and acetyl-CoA production. These metabolites then serve as substrates for p300-mediated histone acetylation, thereby enhancing VEGFR3 expression and promoting lymphangiogenesis (Wong et al., 2017).

Fatty acid uptake and intracellular transport are prerequisites for sustaining FAO. Cifarelli et al. showed that deletion of the fatty acid transporter CD36 in intestinal lymphatic vessels causes lymph leakage in mice, and CD36 knockdown in LECs reduces fatty acid beta-oxidation (Cifarelli et al., 2021). Impaired FAO may therefore damage lymphatic structure and function and contribute to lymphedema progression.

#### 5.1.5 Impaired ketone body oxidation

In addition to glucose and fatty acids, ketone bodies can serve as important metabolic substrates for lymphatic endothelial cells. Ketone body oxidation (KBO) is a major pathway by which the body uses ketone bodies to maintain metabolic homeostasis (Weis et al., 2022). 3-Oxoacid CoA-transferase 1 (OXCT1) and beta-hydroxybutyrate dehydrogenase 1 (BDH1) are key enzymes in KBO (García-Caballero et al., 2019). BDH1 converts beta-hydroxybutyrate into acetoacetate, which is then converted by OXCT1 into acetyl-CoA and enters the TCA cycle to provide energy and metabolic intermediates (Puchalska and Crawford, 2017).

Beyond energy supply, KBO has an important role in regulating lymphatic endothelial function. Garcia et al. found that silencing OXCT1 or BDH1 suppresses KBO and inhibits LEC proliferation, migration, and lymphatic sprouting, whereas beta-hydroxybutyrate promotes these processes (García-Caballero et al., 2019; Weis et al., 2022). Further studies showed that a ketogenic diet improves lymphatic function and reduces immune-cell infiltration in mouse models of lymphedema (García-Caballero et al., 2019). Impaired KBO may therefore restrict LEC function and structure, reduce lymphatic drainage repair capacity, and promote lymphedema progression.

#### 5.1.6 Impaired amino acid transport

Amino acids act as substrates for protein synthesis and can influence the metabolic state and function of LECs by replenishing TCA-cycle intermediates and regulating redox balance. Clinical multi-omics studies have shown reduced levels of branched-chain amino acids, especially valine, and downregulation of the transporter SLC6A15 in adipose tissue from patients with lymphedema, suggesting that reduced branched-chain amino acid availability or impaired transport is a feature of local energy metabolic dysfunction in lymphedema (Karaman et al., 2024).

Guo et al. further revealed how impaired branched-chain amino acid catabolism affects LEC function. Defective branched-chain amino acid degradation causes abnormal intracellular phosphorylation of VEGFR3 and lysosomal degradation, weakening downstream Akt activation and glucose uptake and ultimately suppressing LEC proliferation, migration, and tube formation (Guo et al., 2025). Glutamine also participates in LEC regulation. Huang et al. showed that glutamine provides nitrogen for asparagine synthesis, thereby alleviating endoplasmic reticulum stress and sustaining LEC proliferation and sprouting (Huang et al., 2017). Under hypoxia, glutamine can enhance expression of key glycolytic enzymes and increase lactate production; during persistent hypoxia, however, it may damage lymphatic structure and function (Johandes et al., [[NoYear]]).

The mechanisms of amino acid metabolic abnormalities in lymphedema remain insufficiently defined. Current evidence nevertheless indicates that decreased local branched-chain amino acid levels are a feature of energy metabolic disorder. Impaired branched-chain amino acid catabolism and glutamine-related metabolic abnormalities may reduce lymphatic regeneration by affecting VEGFR3 signaling and glucose utilization, suggesting that amino acid metabolism may represent a new intervention target in lymphedema.

**Figure 2:**
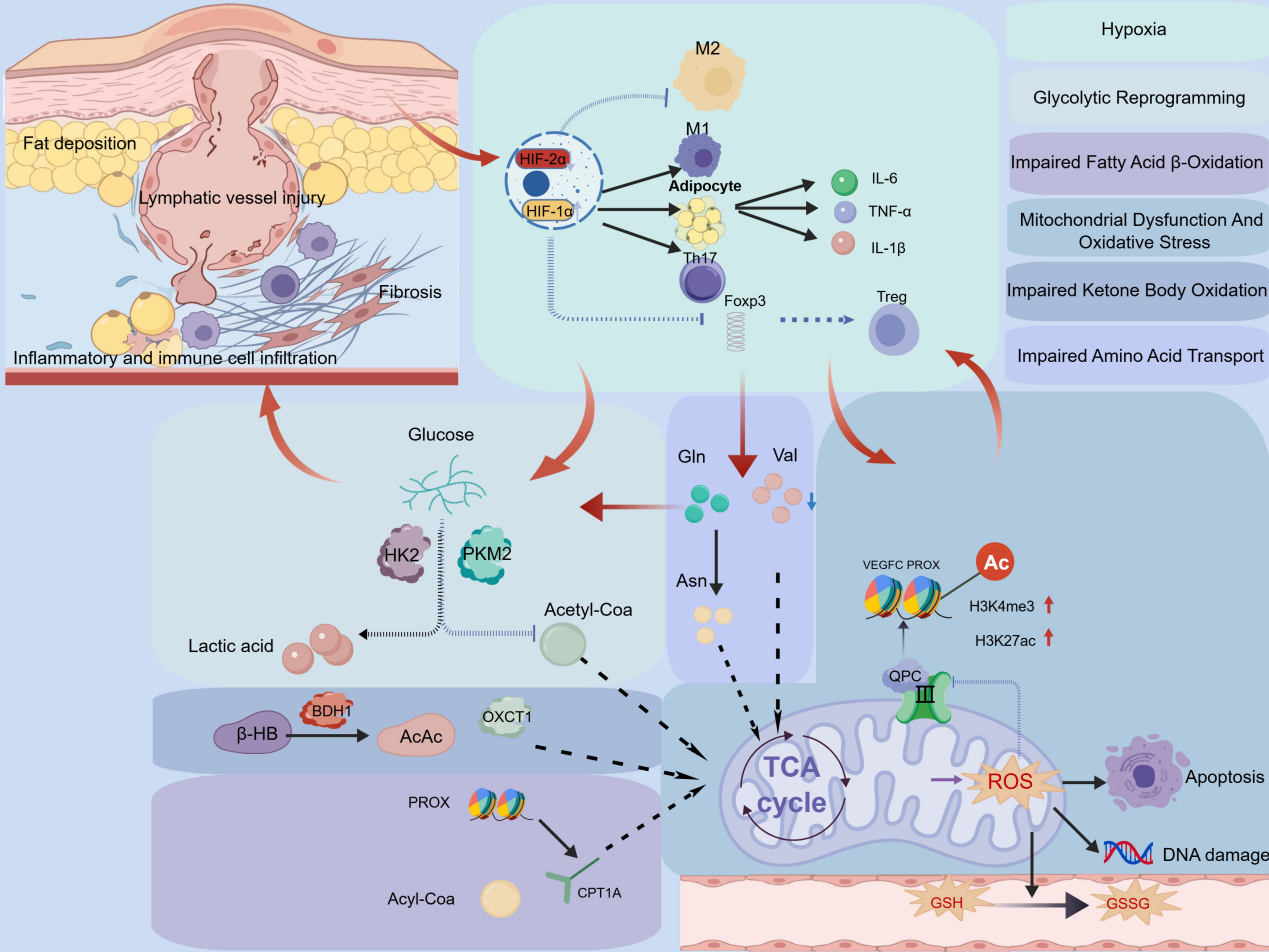
Mechanisms of energy metabolism dysfunction in lymphedema.

In summary, under hypoxic pressure, LECs in lymphedematous tissues exhibit mitochondrial dysfunction and enhanced glycolysis. This metabolic reprogramming may temporarily satisfy cellular energy demands, but lactate accumulation caused by increased glycolysis can further damage the local microenvironment through profibrotic effects. At the same time, loss of mitochondrial membrane potential increases ROS generation, and ROS induces apoptosis and DNA damage, thereby blocking repair of functional lymphatic vessels. The roles of FAO, KBO, and amino acid metabolism in maintaining LEC structure, function, and lymphangiogenesis indicate that lymphatic endothelial metabolism is regulated by multiple substrates in a coordinated manner, although the precise mechanisms require further study (**Figure 2**).

### 5.2 Lipid metabolism dysregulation

#### 5.2.1 Imbalance of fatty acid composition and insulin resistance

Abnormal fatty acid metabolism is an important manifestation of lipid metabolic dysregulation in lymphedema and is closely associated with lymphatic injury and insulin resistance. Studies show that adipose tissue expansion in lymphedema is dominated by adipocyte hypertrophy and accompanied by enhanced lipolysis and reduced insulin sensitivity (Wueest et al., 2009; Longo et al., 2019; Lim et al., 2026). Fatty acids released by lipolysis can damage lymphatic structure and function through lipotoxic effects. Recent clinical studies found a significantly reduced plasma polyunsaturated fatty acid/saturated fatty acid (PUFA/SFA) ratio and increased saturated fatty acid content in patients with secondary lymphedema. *In vitro* experiments further showed that stearic acid induces oxidative stress, endoplasmic reticulum stress, and apoptosis in lymphatic endothelial cells and inhibits their migration and sprouting capacity (Gomes et al., 2025).

Clinical studies currently indicate an association between insulin resistance and lymphedema. Analan et al. reported that patients with lymphedema have significantly higher HOMA-IR indices and fasting insulin levels than controls (Doruk Analan and Kaya, 2022). Excess free fatty acids released from hypertrophic adipocytes and enhanced lipolysis can induce local insulin resistance by activating inflammatory pathways. Specifically, increased free fatty acids can bind Toll-like receptors 2/4 (TLR2/4), activate c-Jun N-terminal kinase (JNK) and NF-kappaB signaling, promote MCP-1 expression, recruit macrophages, and amplify local inflammation (Nguyen et al., 2007). JNK and NF-kappaB signaling can also induce inhibitory serine phosphorylation of IRS1, further aggravating tissue insulin resistance (Aguirre et al., 2002). Reduced insulin sensitivity decreases glucose uptake and mitochondrial substrate supply, damaging lymphatic structure and function and thereby worsening lymphedema. Overall, abnormal fatty acid metabolism promotes disease progression by causing lipotoxic injury to lymphatic endothelial cells and by driving inflammatory signaling-mediated insulin resistance.

#### 5.2.2 Lipid mediator imbalance

Lipid metabolic abnormalities in lymphedema include both fatty acid metabolic disorder and an imbalanced profile of lipid mediators derived from polyunsaturated fatty acids such as arachidonic acid. This imbalance is characterized mainly by suppression of pro-resolving pathways and increased production of pro-inflammatory lipid mediators. 15-Lipoxygenase (15-LO) is constitutively expressed in immune and endothelial cells and catalyzes stereoselective dioxygenation of polyunsaturated fatty acids to generate specialized pro-resolving mediators (SPMs) with anti-inflammatory and tissue-reparative functions (Singh and Rao, 2019). 15-LO is involved in SPM biosynthesis and helps maintain Treg differentiation and pro-resolving function (Marques et al., 2021). Zamora et al. found reduced 15-LO activity in lymphedema, resulting in decreased arachidonic acid (AA)-derived SPMs and suggesting impaired inflammatory resolution (Zamora et al., 2024). In mouse models of lymphedema, inhibition of 15-LO increased CD4+ T cells and Treg apoptosis, aggravated lymphedema, and further demonstrated the protective role of 15-LO (Zamora et al., 2024).

Alongside reduced generation of pro-resolving lipid mediators, arachidonic acid metabolism also shows increased leukotriene (LT) synthesis, indicating a further shift of local lipid mediators toward a pro-inflammatory state. Leukotriene biosynthesis begins when cytosolic phospholipase A2 (cPLA2) releases arachidonic acid (AA) from membrane phospholipids. AA is then converted by 5-lipoxygenase (5-LO) into leukotriene A4 (LTA4). LTA4 can be metabolized by LTA4 hydrolase (LTA4H) to leukotriene B4 (LTB4) or converted in the cytosol by LTC4 synthase (LTC4S) to leukotriene C4 (LTC4). Jiang et al. showed that serum LTB4 levels are significantly elevated in patients with lymphedema (Jiang et al., 2018). LTB4 has concentration-dependent biphasic effects in lymphedema: low levels during early tissue repair can activate VEGFR3 and NOTCH signaling in LECs and promote lymphatic growth (Tian et al., 2017), whereas persistently high levels suppress LEC function, induce apoptosis, and promote lymphedema progression (Murtomaki et al., 2014). Lipid mediator reprogramming in lymphedema therefore aggravates local immune dysregulation, promotes LEC apoptosis, and becomes an important driver of disease progression.

#### 5.2.3 Cholesterol accumulation

The lymphatic system is an important route for peripheral cholesterol clearance and reverse cholesterol transport (RCT) (Randolph and Miller, 2014). A hypercholesterolemic microenvironment can impair lymphatic function. Davis et al. reported that ApoE-/- hypercholesterolemic mice exhibit reduced lymphatic contractility and valve dysfunction (Davis et al., 2023). Local tissue cholesterol accumulation can also damage lymphatic structure and function. Recent studies indicate that local cholesterol accumulation is an important driver of tissue remodeling in lymphedema, with abundant free cholesterol deposits in dermal adipose tissue from patients with secondary lymphedema, accompanied by adipocyte hypertrophy, macrophage infiltration, and fibrosis (Lim et al., 2026; Mehrara, 2026).

Beyond tissue-level cholesterol retention, maintenance of cholesterol homeostasis within lymphatic endothelial cells is also indispensable for lymphatic development and function. Kondo et al. showed that S1P-mediated SREBP2 activation is a key step in cholesterol biosynthesis in LECs (Kondo et al., [[NoYear]]). Cholesterol homeostasis directly affects lymphangiogenesis and repair by maintaining VEGF-C/VEGFR3 signaling and lymphatic endothelial membrane structure and function. Thus, abnormal cholesterol metabolism can manifest as impaired peripheral cholesterol clearance, leading to local cholesterol deposition and promoting adipose remodeling and fibrosis, and can also involve impaired endogenous cholesterol biosynthesis in LECs, downregulating VEGFR3 expression and related signaling and thereby affecting lymphatic structure and function.

#### 5.2.4 Adipokines dysregulation

In lipid metabolic dysregulation in lymphedema, abnormal endocrine function of adipose tissue is also a key component of disease progression. Adipocytes secrete multiple adipokines that directly participate in lymphangiogenesis and local inflammatory regulation (Clemente-Suárez et al., 2023). Adiponectin and leptin are two representative signaling molecules and show distinct regulatory patterns in lymphedema.

Adiponectin appears to have stage-dependent effects in lymphedema. Studies show that adiponectin expression increases after lymphatic drainage is blocked, suggesting a possible role in early inflammatory regulation; however, when adipose tissue becomes hypertrophic and hypoxic, adiponectin expression gradually decreases (Aschen et al., 2012). Shimizu et al. found that adiponectin-knockout lymphedema mice have significantly larger tail diameters than wild-type mice and markedly fewer lymphatic endothelial cells. Mechanistically, adiponectin activates AMPK/Akt/eNOS signaling in LECs, promotes nitric oxide production, and thereby enhances lymphangiogenesis (Shimizu et al., 2013). Adiponectin may therefore contribute both to inflammatory regulation and lymphatic repair.

Leptin regulates the lymphatic system in a concentration-dependent manner. High concentrations of leptin may adversely affect lymphatic endothelial structure and function. Sato et al. reported that human dermal lymphatic endothelial cells express leptin receptors, and high-dose leptin inhibits their proliferation and tube formation through abnormal STAT3 activation and upregulation of the negative feedback regulator SOCS3, while also increasing IL-6 expression and disrupting lymphatic endothelial homeostasis (Sato et al., 2016). Low concentrations of leptin, in contrast, promote LEC proliferation. These findings suggest that physiological leptin levels may help maintain lymphatic endothelial function, whereas high leptin levels can injure lymphatic vessels.

Clinical evidence indicates that adipokine changes in lymphedema may vary by anatomical site. Yusof et al. found that patients with breast cancer-related lymphedema have significantly higher serum leptin levels and a lower adiponectin/leptin ratio than non-lymphedema controls (Yusof et al., 2022). Zaleska et al., however, reported increased serum adiponectin and leptin levels in patients with lower-limb lymphedema (Zaleska and Olszewski, 2017). Because clinical populations are heterogeneous and large-scale clinical studies are lacking, no consistent conclusion has yet been reached regarding adiponectin expression in lymphedema. By comparison, increased leptin and a reduced adiponectin/leptin ratio may more clearly indicate a metabolically inflammatory state in lymphedema.

**Figure 3:**
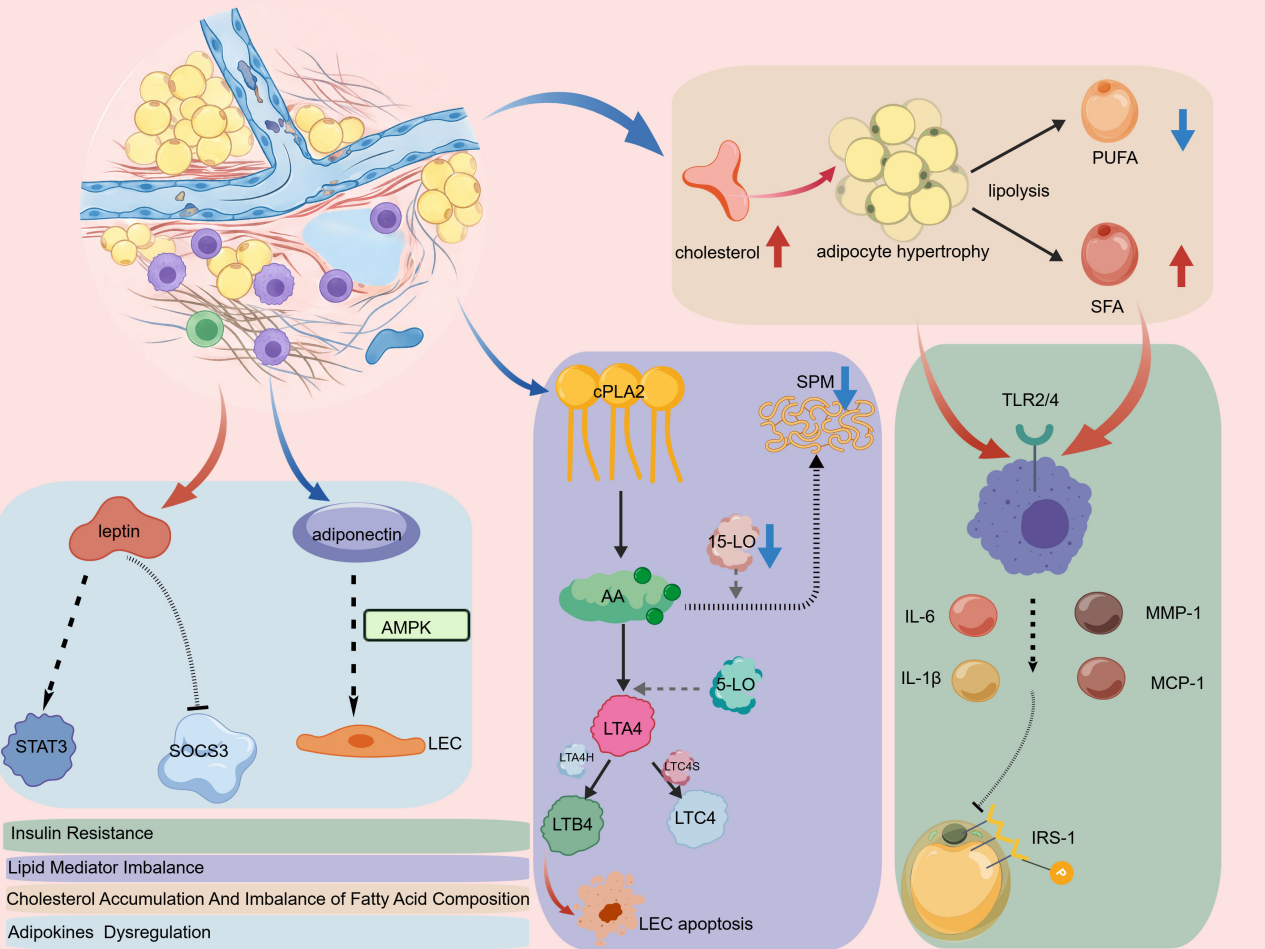
Mechanisms of lipid metabolism dysfunction in lymphedema.

In summary, impaired lymphatic drainage restricts local lipid clearance and promotes adipocyte hypertrophy, enhanced lipolysis, and increased free fatty acids, thereby damaging lymphatic endothelium and amplifying insulin resistance through lipotoxicity and inflammatory signaling. Lipid mediator imbalance is also evident, with attenuation of the 15-LO/SPM pro-resolving pathway and enhancement of the 5-LO/LTB4 pro-inflammatory pathway. Increased leptin and a reduced adiponectin/leptin ratio further participate in inflammatory regulation and impair lymphatic endothelial function. Together, these lipid metabolic disturbances promote lymphedema progression (**Figure 3**).

## 6 Immunometabolic crosstalk in secondary lymphedema

Metabolic abnormalities in secondary lymphedema can remodel the local immune microenvironment through multiple pathways. Hypoxia and HIF signaling enhance glycolysis and influence macrophage polarization, the Th17/Treg balance, and pro-inflammatory cytokine release. Lactate accumulation caused by enhanced glycolysis may promote fibrosis through TGF-beta signaling and epigenetic regulation. Mitochondrial dysfunction and oxidative stress can induce ROS accumulation, DNA damage, and LEC apoptosis, thereby amplifying tissue injury-associated inflammation. In addition, metabolite accumulation caused by impaired lymphatic drainage cannot be overlooked: increased saturated fatty acids, cholesterol deposition, and adipokine abnormalities can activate lipotoxic and inflammatory signaling, further aggravating structural and functional lymphatic damage.

Immune cells are both responders to metabolic abnormalities and participants in reshaping the metabolic microenvironment. In lymphedematous tissues, infiltrating CD4+ T cells, macrophages, neutrophils, and other immune cells are regulated by metabolic signals and also release inflammatory and fibrotic mediators, including TNF-alpha, IL-6, IL-1beta, and TGF-beta. These factors in turn promote adipose expansion, extracellular matrix deposition, and lymphatic dysfunction. Thus, metabolic abnormalities in sec-ondary lymphedema affect the energy supply and structural ho-meostasis of lymphatic endothelial cells and continuously drive inflammation, adipose deposition, and fibrosis by regulating immune-cell infiltration, macrophage polarization, the Th1/Th2/ Th17/Treg balance, and inflammatory cytokine release (**Table 1**).

**Table 1:** Metabolic pathways and immune consequences in secondary lymphedema.

Metabolic abnormality	Metabolic mechanism or clinical finding	Immune changes	Outcome	Evidence level	References
Hypoxia	Under hypoxia, glucose uptake and glycolytic flux increase; pyruvate dehydrogenase is inhibited, blocking conversion of pyruvate to acetyl-CoA for TCA-cycle entry and promoting lactate generation.	M1-like macrophage polarization, increased pro-inflammatory cytokine release, and a Th17-skewed Th17/Treg balance.	Early responses may maintain basic energy needs under hypoxia; chronic persistence can enhance glycolysis and cause lactate accumulation.	Level 2, Level 4	(Dang et al., 2011; Lee et al., 2020)
Hypoxia	Early hypoxia may induce lymphangiogenic signals such as HIF-1alpha-mediated VEGF-C/VEGFR3 and participate in reparative lymphangiogenesis; persistent hypoxia may involve sustained HIF-1alpha elevation, relative HIF-2alpha insufficiency, adipocyte death, insulin resistance, and local metabolic inflammation.	Increased CD4+ T cells, including Th1, Th2, Th17, and Treg cells; Th2 cells promote fibrosis, while Th1 and Th17 cells promote nonfunctional lymphangiogenesis. Hypoxia can recruit macrophages; HIF-1alpha promotes M1-like macrophage responses, Th17 differentiation, and FOXP3+ Treg instability, whereas HIF-2alpha contributes to some M2-like macrophage responses and helps maintain Treg stability and suppressive function.	Early hypoxia may promote reparative lymphangiogenesis; persistent hypoxia can aggravate adipose inflammation, insulin resistance, and impaired inflammatory resolution, induce nonfunctional lymphangiogenesis, and ultimately worsen lymphatic dysfunction.	Level 2, Level 3, Level 4	(Yin et al., 2009; Imtiyaz et al., 2010; Dang et al., 2011; Min et al., 2011; Mazzatti et al., 2012; Zampell et al., 2012a; Fujisaka et al., 2013; García-Martín et al., 2016; Hsu et al., 2020)
Glycolytic reprogramming	Enhanced glycolysis under hypoxia increases lactate production and reduces pyruvate entry into mitochondrial oxidation; lactate can lower local pH, activate TGF-beta, and participate in histone lactylation.	Lactate-derived histone lactylation can induce M2-like or repair-related genes such as Arg1 and Vegfa during the late phase of M1 macrophage polarization.	Promotes fibroblast-to-myofibroblast differentiation, ECM deposition, and fibrotic progression.	Level 4	(Xie et al., 2015; Zhang et al., 2019; Jiang et al., 2021)
Mitochondrial dysfunction and oxidative stress	Plasma GSH decreases, while GSSG, MDA, and 4-HNE increase.	–	Redox imbalance and lipid peroxidation may aggravate lymphatic endothelial injury, tissue inflammation, and fibrotic remodeling.	Level 1	(Siems, 2002; Yang et al., 2021)
Mitochondrial dysfunction and oxidative stress	Oxidative injury and VEGF-C levels are increased in mouse tail lymphedema; in LECs, VEGF-C/VEGFR3 signaling under H2O2-induced oxidative stress enhances ROS generation, DNA damage, mitochondrial dysfunction, and LEC apoptosis.	Elevated VEGF-C levels are associated with macrophage infiltration.	Under oxidative stress, VEGF-C shifts from a pro-lymphangiogenic signal to a pro-injury signal in LECs, reducing lymphatic repair capacity and impairing recovery of lymphatic drainage.	Level 2, Level 3	(Hossain et al., 2024)
Mitochondrial dysfunction and oxidative stress	Mitochondrial injury causes loss of membrane potential, increased ROS, and DNA damage; ROS further impairs mitochondrial function.	–	–	Level 4	(Turrens, 1997)
Impaired fatty acid beta-oxidation	CD36 deletion in intestinal lymphatics causes lymph leakage, and CD36 knockdown in LECs reduces FAO.	–	Impaired lymphatic structure and function.	Level 2, Level 3	(Cifarelli et al., 2021)
Impaired ketone body oxidation	A ketogenic diet increases ketone body availability.	Reduced infiltration of TCRbeta+ CD4+ T cells and TCRbeta+ CD8+ T cells.	Promotes formation of newly generated functional lymphatic vessels in tail tissues of lymphedema mice.	Level 2	(García-Caballero et al., 2019)
Impaired ketone body oxidation	A ketogenic diet increases ketone body availability.	–	Restoration of lymphatic function and reduced dermal backflow in patients with secondary lymphedema.	Level 1	(Lodewijckx et al., 2024)
Impaired amino acid transport	Branched-chain amino acids, especially valine, decrease in lymphedema adipose tissue, with downregulation of SLC6A15.	–	–	Level 1	(Karaman et al., 2024)
Impaired amino acid transport	Impaired BCAA degradation causes abnormal VEGFR3 phosphorylation and lysosomal degradation, weakening Akt activation and glucose uptake.	–	–	Level 4	(Guo et al., 2025)
Impaired amino acid transport	Glutamine provides nitrogen for asparagine synthesis, relieves endoplasmic reticulum stress, and supports LEC proliferation and sprouting; under hypoxia, glutamine can enhance glycolysis and lactate generation.	–	Moderately supports LEC growth; during persistent hypoxia, it may impair lymphatic structure and function.	Level 3, Level 4	(Johandes et al., [[NoYear]]; Huang et al., 2017)
Imbalance of fatty acid composition and insulin resistance	The plasma PUFA/SFA ratio decreases; increased saturated fatty acid load induces ER stress, oxidative stress, mitochondrial ROS generation, and apoptosis in LECs.	Increased perilymphatic F4/80+ macrophage and CD4+ T-cell infiltration.	LEC lipotoxic injury, impaired lymphatic repair, and progression of secondary lymphedema.	Level 1, Level 2, Level 3	(Gomes et al., 2025)
Imbalance of fatty acid composition and insulin resistance	Adipocyte hypertrophy and enhanced lipolysis increase free fatty acid release; FFAs activate TLR2/4 and JNK/NF-kappaB-related inflammatory pathways, and JNK1 induces IRS-1 Ser307 phosphorylation, blocking IRS-1 binding to the insulin receptor.	FFAs induce upregulation of IL-1beta, IL-6, MCP-1, TNF, and MMP-9 and promote macrophage recruitment.	Lipotoxicity and chronic inflammation may impair local lymphatic structure and function.	Level 4	(Nguyen et al., 2007)
Imbalance of fatty acid composition and insulin resistance	Patients with lymphedema have significantly higher BMI, waist circumference, serum insulin, and HOMA-IR than non-BCRL breast cancer controls.	–	–	Level 1	(Doruk Analan and Kaya, 2022)
Lipid mediator imbalance	15-LO activity and 15-LO-derived SPMs decrease; AA-, DHA-, and EPA-derived pro-resolving lipid mediators are reduced.	Increased CD3+/CD4+ T-cell infiltration; reduced PPARgamma+ Tregs or increased apoptotic Tregs.	Reduced inflammatory-resolution capacity, persistent chronic inflammation, lymphatic network disruption, aggravated edema, and promotion of adipose deposition and fibrosis.	Level 1, Level 2	(Marques et al., 2021; Zamora et al., 2024)
Lipid mediator imbalance	Arachidonic acid is converted by 5-LO to LTA4 and then by LTA4H to LTB4.	Recruitment of neutrophils, macrophages, and T cells, with links to Th17 differentiation and inflammatory amplification.	Enhanced inflammation and induction of LEC apoptosis.	Level 1, Level 2, Level 3	(Tian et al., 2017)
Cholesterol accumulation	Free cholesterol deposits in dermis and around lymphatic vessels.	CD68+ macrophages form crown-like structures around adipocytes, and CD3+ T-cell infiltration increases.	Dermal adipose remodeling, adipocyte hypertrophy and death, collagen deposition, and fibrosis.	Level 1, Level 2	(Lim et al., 2026; Mehrara, 2026)
Cholesterol accumulation	ApoE-/- hypercholesterolemic mice show reduced lymphatic contractility and valve dysfunction.	Increased dermal CD45+ leukocyte infiltration.	Reduced lymphatic pump function, valve dysfunction, and lower lymphatic drainage efficiency.	Level 2	(Davis et al., 2023)
Adipokine dysregulation	Adiponectin deficiency or weakened adiponectin signaling reduces AMPK/Akt/eNOS signaling.	–	Reduced lymphatic repair capacity.	Level 2, Level 3	(Shimizu et al., 2013)
Adipokine dysregulation	Patients with lymphedema have increased serum leptin; leptin can induce SOCS3 upregulation through JAK/STAT3 signaling, impairing HDLEC proliferation and tube formation while promoting IL-6 expression.	–	Disrupted LEC homeostasis and impaired lymphangiogenesis.	Level 1, Level 3	(Sato et al., 2016; Yusof et al., 2022)
Adipokine dysregulation	Adipogenic markers such as PPARgamma, C/EBPalpha, and adiponectin are upregulated.	–	Subcutaneous adipose deposition.	Level 1	(Aschen et al., 2012)

Evidence level: Level 1 = direct human lymphedema evidence; Level 2 = preclinical lymphedema model evidence; Level 3 = LEC-based or *in vitro* evidence; Level 4 = extrapolated evidence.

## 7 Obesity and lifestyle modulation of the lymphedema metabolic microenvironment

Obesity is a systemic metabolic disorder characterized by abnormal adipose tissue expansion, chronic low-grade inflammation, and metabolic dysfunction, and it is an important pathological basis for the development and aggravation of lymphedema. Obesity is closely associated with an increased risk of secondary lymphedema; obese patients undergoing breast cancer surgery have a significantly higher risk of secondary lymphedema than non-obese patients (Shaw et al., 2007a). Some patients with severe obesity also develop abnormal lymphoscintigraphy findings and clinical lymphedema even without a definite history of surgery, radiotherapy, or trauma, indicating that obesity itself can induce lymphatic functional decompensation (Sudduth and Greene, 2022).

Studies have shown that high-fat diet (HFD)-induced obese mice exhibit decreased dermal lymphatic vessel density, reduced LEC proliferation, and increased lymphatic permeability (Blum et al., 2014; García Nores et al., 2016), demonstrating that obesity can directly impair lymphatic structure and function. Obesity also affects immune transport by the lymphatic system, as reflected by reduced dendritic-cell migration to lymph nodes, abnormal lymphatic structure, and decreased lymph node size (Weitman et al., 2013). Chronic inflammation is a key mechanism by which obesity promotes lymphedema progression. Under conditions of energy surplus, hypertrophic adipocytes fail to maintain normal metabolic homeostasis and release pro-inflammatory mediators such as TNF-alpha, IL-6, and MCP-1, thereby promoting macrophage infiltration and amplifying inflammation (Longo et al., 2019). With persistent inflammation, CD11b+ macrophages, iNOS+ macrophages, and CD4+ T lymphocytes can accumulate around lymphatic vessels, creating a pathological microenvironment characterized by perilymphatic inflammation (Mraz and Haluzik, 2014). This inflammatory response can damage lymphatic structure and barrier function, aggravating lymph leakage (Schwager and Detmar, 2019). Lipotoxicity is another important mechanism by which obesity impairs lymphatic function. In obesity, lipid concentrations and free fatty acid profiles are altered, and increased saturated fatty acid burden can directly injure LECs (Gomes et al., 2025). Lipotoxicity can induce endothelial dysfunction, increase oxidative stress, and reduce nitric oxide production. As NO is an important regulator of lymphatic contractility and tone (Liao et al., 2011), reduced NO can decrease lymphatic pump efficiency (Ohhashi et al., 2023).

Lifestyle intervention targets obesity-induced immunometabolic dysregulation and is therefore one of the most clinically feasible non-pharmacologic strategies for lymphedema management. Weight control is a key foundation for improving the microenvironment of obesity-related lymphedema. A randomized controlled study by Shaw et al. showed that weight loss through reduced energy intake significantly reduces affected-limb volume in patients with breast cancer-related lymphedema (Shaw et al., 2007a). Another randomized controlled study comparing a low-fat diet with a weight-reduction diet showed that both regimens reduced body weight, BMI, and skinfold thickness. Although the between-group difference in excess limb volume did not reach significance, weight loss correlated significantly with reduced excess limb volume, suggesting that the main benefit of a low-fat diet derives from weight reduction and lower adipose burden (Shaw et al., 2007b). Beyond energy restriction alone, specific dietary patterns may influence the lymphedema microenvironment by regulating substrate utilization and inflammation. In obese patients with breast cancer-related lymphedema, Vafa et al. conducted a randomized controlled trial and found that a low-calorie diet combined with synbiotics improved quality of life, edema volume, and BMI (Vafa et al., 2020). More recently, ketogenic diets have provided a metabolic perspective for dietary treatment of lymphedema. Lodewijckx et al. demonstrated that a ketogenic diet can improve lymphatic function in patients with secondary lymphedema, although its effects on limb edema volume remain inconsistent (Lodewijckx et al., 2024). Polyphenols in olive oil may also act by regulating inflammatory lipid mediators. Hydroxytyrosol, one of the major phenolic compounds in olive oil, has anti-inflammatory effects and reduces LTB4 synthesis (Bertelli et al., 2020). Olive oil intake has also been associated with improved quality of life in patients with primary lymphedema (Improvement of quality of life by intake of hydroxytyrosol in patients with lymphedema and association of lymphedema genes with obesity).

Compared with dietary intervention, exercise has the dual effects of promoting lymphatic return and improving systemic metabolic status and is another key component of lifestyle intervention. Patients after breast cancer surgery were historically advised to avoid upper-limb loading to prevent the onset or worsening of lymphedema (Ahmed et al., 2006). Subsequent randomized studies gradually showed that progressive resistance training under appropriate supervision is generally safe and may improve lymphedema symptoms (Schmitz et al., 2009). Mechanistically, exercise may affect lymphedema through both mechanical and metabolic pathways. Mechanically, rhythmic skeletal muscle contraction enhances the muscle pump, promotes venous and lymphatic return, and may improve interstitial fluid clearance under compression therapy. Metabolically, regular exercise reduces fat mass, improves insulin sensitivity and lipid profiles, alleviates obesity-related chronic low-grade inflammation, and improves vascular endothelial function. For patients with secondary lymphedema, exercise should therefore be understood as a comprehensive intervention that regulates host metabolic status, lowers inflammatory burden, and improves lymphatic dynamics.

Exercise is another lifestyle intervention that affects both lymphatic dynamics and metabolic status. Skeletal muscle contraction enhances the muscle pump and promotes venous and lymphatic return; long-term exercise can also reduce adipose inflammation, improve insulin sensitivity, and regulate obesity-related metabolic abnormalities (Hasenoehrl et al., 2020). In an animal study, Hespe et al. showed that aerobic exercise, even without substantial weight loss, reduced perilymphatic inflammatory-cell accumulation, improved lymphatic function, and reversed pathological LEC gene-expression changes involving VEGFR3 and PROX1 (Hespe et al., 2016). The benefits of exercise in lymphedema therefore involve both local inflammation relief and restoration of lymphatic endothelial and lymphatic pump function.

Overall, obesity shapes an immunometabolically imbalanced microenvironment in lymphedema through lymphatic structural injury, chronic inflammation, and lipotoxicity. Lifestyle interventions can reduce adipose burden, improve local inflammation, and optimize metabolic substrate utilization, providing a practical strategy for targeting the immunometabolic microenvironment of lymphedema.

## 8 Targeted drugs for the metabolic microenvironment in lymphedema and supporting evidence

Targeting the local metabolic microenvironment is emerging as a new therapeutic strategy for lymphedema. Multiple metabolic modulators have shown protective effects on the lymphatic system and local tissues in animal models or clinical studies. Their core mechanisms include energy metabolic remodeling, alleviation of lipotoxicity, restoration of redox homeostasis, and targeted anti-inflammatory effects. Major drug classes include AMPK pathway agonists, PPARgamma agonists, GLP-1 receptor agonists, cholesterol-depleting agents, nonsteroidal anti-inflammatory drugs, and selenium supplementation.

### 8.1 AMPK pathway agonists

Metformin is a classical metabolic regulator that activates the AMP-activated protein kinase (AMPK) pathway and is widely used in type 2 diabetes and other metabolic diseases (Luo et al., 2016). Beyond improving insulin resistance and regulating glucose and lipid metabolism, metformin can also influence inflammation and oxidative stress (Wei et al., 2025). A recent retrospective clinical analysis of breast cancer-related lymphedema suggested that metformin use is associated with a lower risk of breast cancer-related lymphedema (Rubin et al., 2025), providing preliminary clinical evidence for its potential application. Further animal studies showed that metformin reduces limb and tail swelling in lymphedema mice by decreasing CD4+ T-cell numbers, suppressing Th2- and Th17-derived cytokines, and downregulating IL-4, IL-13, IL-17, and TGF-beta1 expression (Wei et al., 2024).

In addition to modulating inflammation and fibrosis, metformin exerts therapeutic effects on the metabolic microenvironment by improving mitochondrial function and reducing oxidative stress. Yang et al. showed that metformin improves mitochondrial function by increasing membrane potential, reducing ROS and MDA levels, and increasing SOD activity (Yang et al., 2022). Hartwig et al. further found that metformin can upregulate antioxidant molecules such as manganese superoxide dismutase (SOD2) through AMPK-dependent activation of forkhead box O3 (FOXO3), thereby reducing ROS levels (Hartwig et al., 2021). Metformin may therefore improve the lymphedema microenvironment by regulating inflammation, fibrosis, oxidative stress, and other processes. However, apart from limited retrospective clinical studies and animal experiments, higher-level human evidence remains insufficient, and its efficacy and safety require validation in prospective clinical studies.

### 8.2 PPARγ agonists

PPARgamma agonists have traditionally been used to treat type 2 diabetes and insulin resistance. Because they regulate lipid distribution, reduce lipotoxicity, and maintain metabolic homeostasis, these agents have recently been considered to have therapeutic potential in lymphedema. Rosiglitazone, a representative drug, promotes lipid uptake into adipose tissue and lowers free fatty acid levels, thereby improving the local tissue microenvironment. Chen et al. showed that rosiglitazone reduces skin fibrosis and adipose deposition in mice with secondary lymphedema. *In vitro* experiments further indicated that rosiglitazone regulates the phenotype of PDGFRalpha+ adipose-derived stem cells and upregulates adipogenic gene expression, thereby slowing fibroadipose tissue remodeling (Chen et al., 2023).

The metabolic benefits of PPARgamma agonists in lymphedema are mainly reflected in two aspects. By promoting lipid uptake and storage in adipose tissue and lowering free fatty acid levels, PPARgamma agonists can alleviate lipotoxic injury to local cells and inhibit free fatty acid-triggered inflammatory responses and insulin resistance mediated through TLR2/4 and JNK pathways (Ye et al., 2004; Shi et al., 2006). PPARgamma signaling is also important for maintaining lymphatic endothelial structure and function. Morris et al. showed that impaired lymphatic flow can upregulate Krüppel-like factor 2 (KLF2), inhibit PPARgamma signaling in LECs, increase ROS, and ultimately damage lymphatic endothelial function (Morris et al., 2018). Overall, rosiglitazone may act as a metabolic intervention by reducing lipotoxicity, suppressing fibroadipose tissue remodeling, and improving the inflammatory microenvironment. However, rosiglitazone can increase renal sodium and water reabsorption and cause fluid retention and peripheral edema (Panchapakesan et al., 2009), so its clinical use in lymphedema requires further evaluation.

### 8.3 GLP-1 receptor agonists

Semaglutide is a GLP-1 receptor agonist (GLP-1RA) that has mainly been used for obesity treatment. Recent studies suggest that GLP-1RA therapy may improve lymphedema symptoms. A case report by Crowley et al. described a patient with breast cancer-related lymphedema whose affected-limb volume decreased and body weight declined after GLP-1RA treatment (Crowley et al., 2024). Brown et al. further found similar clinical improvement in a retrospective cohort of patients after axillary lymph node dissection, including 76 patients who received GLP-1RA therapy (Brown et al., 2024).

GLP-1 receptor agonists (GLP-1RAs) may exert therapeutic effects by improving the metabolic environment, reducing inflammation and fibrosis, and regulating lymphatic function. In metabolic regulation, GLP-1RAs promote glucose-dependent insulin secretion and beta-cell survival through cAMP/PKA/CREB signaling, while also activating the PI3K/AKT pathway to enhance peripheral glucose uptake and improve insulin resistance, which may help restore lymphatic endothelial function (Akl and Ahmed, 2025). GLP-1RAs can also activate eNOS, increase NO production, and reduce oxidative stress, thereby protecting lymphatic endothelial structure and improving the local microenvironment (Ding and Zhang, 2012).

With respect to anti-inflammatory and anti-fibrotic effects, GLP-1RAs reduce chronic inflammation and tissue injury by suppressing inflammatory mediator release, downregulating TLR4 signaling, and reducing CD4+ T cell-associated inflammatory responses and cytokines such as IL-5 (Toki et al., 2021). These drugs may also reduce accumulation of advanced glycation end products (AGEs), inhibit receptor for advanced glycation end products (RAGE)-mediated inflammation, and downregulate profibrotic factors such as TGF-beta, thereby slowing lymphedema progression (Taguchi and Fukami, 2023). In addition to these metabolic and inflammatory effects, GLP-1RAs may directly regulate lymphatic function. Schulz et al. showed that GLP-1RA therapy restores lymphatic contractility in obese and ApoE-deficient mice (Schulz et al., [[NoYear]]). Most mechanistic evidence, however, still comes from non-lymphedema models, and the metabolic mechanisms of GLP-1RAs in lymphedema require further validation.

### 8.4 Cholesterol-depleting agents

Targeting cholesterol deposition has become a potential metabolic intervention for lymphedema. Several drugs that reduce cholesterol accumulation have been widely studied in atherosclerosis, Niemann-Pick disease, and related disorders (Davis et al., 2001; Lopez et al., 2014), and some have begun to show therapeutic potential in animal models of lymphedema. Statins reduce endogenous cholesterol synthesis by inhibiting HMG-CoA reductase; ezetimibe reduces exogenous cholesterol entry into the circulation by acting on NPC1L1 (Shirouchi et al., 2012); and cyclodextrins, such as hydroxypropyl-beta-cyclodextrin, can directly bind and remove tissue cholesterol (Trasca et al., 2026).

In animal models of lymphedema, cyclodextrin clears locally retained cholesterol and promotes lymphatic regeneration and drainage recovery (Lim et al., 2026; Mehrara, 2026). Its mechanism extends beyond cholesterol removal and may involve inhibition of cholesterol crystal uptake by monocytes/macrophages, promotion of cholesterol efflux, and attenuation of inflammation. Studies show that cyclodextrin reduces cholesterol crystal phagocytosis by circulating monocytes and may promote a macrophage phenotype more favorable for cholesterol metabolism and inflammatory resolution through LXR-ABCA1/ABCG1-related programs (Lübbering et al., 2025). Atorvastatin, although it does not directly remove cholesterol, can inhibit pathological lymphangiogenesis and tissue edema, potentially by suppressing Th1 and Th17 activation and reducing macrophage VEGF-C expression, indicating that cholesterol metabolism interventions and immune regulation may be coupled in lymphedema (Ogata et al., 2016). Overall, current evidence for direct cholesterol-depleting therapy in lymphedema remains mainly animal-based, and its efficacy and safety require further validation in human tissues.

### 8.5 Nonsteroidal anti-inflammatory drugs

Ketoprofen is a nonsteroidal anti-inflammatory drug (NSAID) traditionally regarded as exerting anti-inflammatory, analgesic, and antipyretic effects by inhibiting cyclooxygenase (COX) and blocking prostaglandin synthesis. Recent studies have found that ketoprofen can also inhibit 5-lipoxygenase (5-LO), thereby interfering with leukotriene and other lipid mediator production in the arachidonic acid pathway. This property gives ketoprofen potential value in metabolism-oriented lymphedema therapy.

Nakamura et al. showed that ketoprofen reduces tail swelling, improves epidermal thickening, and suppresses inflammation in lymphedema mice, while increasing expression of lymphatic repair-related molecules including VEGF-C, VEGFR-3, and Prox1 (Nakamura et al., 2009). Based on these preclinical data, Rockson et al. conducted clinical studies of ketoprofen in lymphedema. In a 21-patient study, four months of ketoprofen treatment significantly reduced affected-limb skin thickness; a subsequent randomized controlled study similarly showed reduced skin thickness and decreased plasma granulocyte colony-stimulating factor expression (Rockson et al., 2018). These findings suggest that ketoprofen may act in lymphedema by inhibiting 5-LO activity, reducing inflammatory lipid mediators such as leukotrienes, and promoting lymphatic repair and regeneration. As an NSAID, however, ketoprofen is commonly associated with gastrointestinal and cardiovascular adverse effects, limiting the safety of long-term use.

### 8.6 Selenium supplementation

Sodium selenite provides selenium for selenoprotein synthesis, including glutathione peroxidase (GPX) and thioredoxin reductase (TrxR), and was therefore initially considered a drug that could improve oxidative stress in lymphedema (Han et al., 2019). Previous studies indicated that ROS production increases in lymph-stasis tissues and impairs lymphatic contraction and drainage, while sodium selenite supplementation may alleviate lymphedema symptoms by enhancing selenoenzyme-related redox regulation (Zimmermann et al., 2005). Zimmermann et al. observed that sodium selenite treatment was associated with symptom improvement in patients with postoperative head and neck secondary lymphedema. Tissue edema severity was negatively correlated with serum selenium levels and GPX activity and positively correlated with ROS levels (Zimmermann et al., 2005). Kasseroller et al. further showed that sodium selenite combined with complex decongestive therapy reduces limb edema and lowers the incidence of complications such as erysipelas (Kasseroller and Schrauzer, 2000). Micke and Bruns et al. also observed similar clinical improvements in patients with post-radiotherapy head and neck edema (Micke et al., 2003; Bruns et al., 2004).

Recent randomized controlled evidence indicates that although sodium selenite can improve clinical staging, classical oxidative stress markers such as GSH and GSSG do not change significantly, suggesting that its therapeutic effect in lymphedema may involve non-antioxidant pathways. Current evidence more strongly supports sodium selenite as a potential adjunct to complex decongestive therapy, with mechanisms that may involve redox regulation, anti-inflammatory effects, and immune modulation (Lee et al., 2021). For example, sodium selenite may inhibit NF-kappaB activation and downregulate TNF-alpha-induced adhesion molecules such as ICAM-1, VCAM-1, and E-selectin, thereby reducing inflammatory-cell adhesion and local inflammation and improving the tissue microenvironment (Pfister et al., 2016). Overall, existing studies support sodium selenite as an adjunctive metabolic therapy in addition to complex decongestive treatment, but its optimal indications, dosing regimen, and precise mechanisms remain to be defined.

In summary, therapies targeting the metabolic microenvironment offer new interventional concepts for lymphedema. These agents may exert therapeutic effects through multiple mechanisms, including improving energy metabolic abnormalities, alleviating lipotoxicity, reducing oxidative stress, regulating inflammation and fibrosis, and promoting lymphangiogenesis, indicating that the metabolic microenvironment is a clinically relevant therapeutic entry point (**Table 2**).

**Table 2:** Metabolism-targeted drugs for lymphedema and the supporting evidence.

Drug class	Representative agents	Metabolic mechanisms and clinical findings	Evidence level (model/population)	References
AMPK pathway agonist	Metformin	Metformin was associated with a reduced postoperative lymphedema risk after axillary lymph node dissection.	Patients with breast cancer, diabetes, and lymphedema (n=407)	(Rubin et al., 2025)
AMPK pathway agonist	Metformin	Metformin reduced CD4+ T-cell infiltration and collagen I deposition and increased the number of lymphatic vessels.	Mouse lymphedema model (n=15)	(Wei et al., 2024)
PPARgamma agonist	Rosiglitazone	Rosiglitazone regulated the phenotype of PDGFRalpha+ adipose-derived stem cells, restored adipogenic gene expression, and reduced adipose deposition and fibrosis.	Mouse lymphedema model (n=32)	(Chen et al., 2023)
Cholesterol-depleting agent	Cyclodextrin	Cyclodextrin-mediated cholesterol removal reduced limb swelling, promoted lymphangiogenesis, and restored drainage function.	Mouse lymphedema model (n=17); APOE-knockout mice (n=73)	(Lim et al., 2026)
Cholesterol-depleting agent	Atorvastatin	Atorvastatin inhibited Th1/Th17 activation and reduced macrophage VEGF-C expression.	Mouse lymphedema model (n=20)	(Ogata et al., 2016)
GLP-1 receptor agonist	Semaglutide	After semaglutide therapy, the limb volume difference in a patient with breast cancer-related lymphedema decreased from 10.3% to 3.4%.	Patient with breast cancer-related lymphedema (n=1)	(Crowley et al., 2024)
GLP-1 receptor agonist	Semaglutide	Use of GLP-1RA after axillary lymph node dissection was associated with a significantly lower incidence of lymphedema.	Patients after axillary lymph node dissection (n=3,830; 76 treated with GLP-1RA)	(Brown et al., 2024)
NSAID	Ketoprofen	Ketoprofen reduced skin thickness and inflammatory histological changes and upregulated lymphatic repair-related molecules including VEGF-C, VEGFR-3, and Prox1.	Mouse lymphedema model (n=40)	(Nakamura et al., 2009)
NSAID	Ketoprofen	Ketoprofen significantly reduced affected-limb skin thickness and decreased 5-LO enzymatic activity.	Patients with lymphedema (n=55)	(Rockson et al., 2018)
Selenium supplementation	Sodium selenite	Sodium selenite therapy improved clinical stage in patients with BCRL.	Patients with breast cancer-related lymphedema (n=26)	(Han et al., 2019)
Selenium supplementation	Sodium selenite	Sodium selenite reduced lymphedema symptoms after oral tumor surgery; edema severity was negatively correlated with serum selenium concentration and GPX activity.	Patients with lymphedema after oral squamous cell carcinoma surgery (n=20)	(Zimmermann et al., 2005)
Selenium supplementation	Sodium selenite	Sodium selenite combined with complex decongestive therapy reduced affected-limb volume by 52% and decreased the incidence of erysipelas.	Patients with breast cancer-related lymphedema (n=179)	(Kasseroller and Schrauzer, 2000)
Selenium supplementation	Sodium selenite	After 4 to 6 weeks of oral sodium selenite, VAS scores significantly improved, most patients improved by at least one clinical stage, upper-limb circumference decreased, and some patients with internal laryngeal edema avoided tracheotomy.	Patients with upper-limb lymphedema (n=12) and head and neck lymphedema (n=36)	(Bruns et al., 2004)
Selenium supplementation	Sodium selenite	Intravenous sodium selenite reduced clinical symptoms and decreased the upper-limb ECW/SW ratio; metabolomics showed reprogramming of lipid, nucleotide, and vitamin metabolism and increased anti-inflammatory metabolites.	Patients with breast cancer-related lymphedema (n=29)	(Lee et al., 2021)

## 9 Limitation

Although increasing evidence supports the view that secondary lymphedema is accompanied by local metabolic dysfunction, the disease itself shows substantial clinical heterogeneity. This heterogeneity makes it difficult to extrapolate findings from one specific subtype to all forms of secondary lymphedema. The clinical heterogeneity of secondary lymphedema is mainly reflected in three dimensions: etiology, anatomical site, and disease stage. Lymphatic transport failure caused by surgery, radiotherapy, infection, trauma, or cancer treatment may generate distinct local microenvironments. In addition, upper-limb, lower-limb, and head and neck lymphedema differ in lymphatic anatomy and local tissue architecture. The biological significance of metabolic pathways may also change as the disease progresses. Metabolic pathways that exert compensatory or protective effects in the early stage may become pathological drivers of disease deterioration during the chronic or advanced phases.

At present, mechanistic evidence for local metabolic dysregulation in secondary lymphedema is unevenly distributed across different metabolic pathways. Metabolic processes such as oxidative stress, amino acid metabolic disturbance, fatty acid metabolic imbalance, cholesterol deposition, peripheral lipid mediator dysregulation, and adipokine disturbance have been supported by evidence from human tissues, multi-omics analyses, or clinical cohorts. By contrast, conclusions regarding energy metabolic remodeling, including glycolytic reprogramming, fatty acid β-oxidation, ketone body oxidation, mitochondrial dysfunction, and hypoxia in lymphatic endothelial cells, remain largely dependent on animal models, *in vitro* cell experiments, or extrapolations from other metabolic disease models such as obesity and diabetes. Direct evidence of energy metabolic dysfunction from human lymphedematous tissues remains relatively limited.

Finally, caution is also warranted when evaluating metabolism-targeted interventions that act on the local microenvironment. Metformin, PPARγ agonists, GLP-1 receptor agonists, cholesterol-clearing agents, ketoprofen, and selenium supplementation have shown mechanistic relevance or early translational potential. However, the level of evidence varies considerably among these interventions, ranging from animal experiments and *in vitro* studies to retrospective analyses, case reports, and small clinical cohorts. Prospective, large-scale randomized controlled trials remain very limited, and no metabolism-targeted pharmacological therapy has yet been formally established as a standard clinical treatment for lymphedema. Future studies should therefore further evaluate the safety, long-term efficacy, optimal timing, and most suitable target populations for local metabolism-oriented therapies.

## 10 Future perspectives

In recent years, local metabolic dysfunction has increasingly emerged as an important perspective for understanding the progression of secondary lymphedema, although its precise role remains to be further defined. Future studies should clarify the pathological significance of metabolic abnormalities at different disease stages, with particular attention to distinguishing early adaptive metabolic responses from decompensated metabolic injury during the chronic phase.

At the mechanistic level, future research should establish secondary lymphedema models that more closely recapitulate clinical conditions, such as composite models combining surgical lymphatic injury with radiotherapy, obesity, diabetes, or chronic inflammation. Such models may more accurately reflect the immunometabolic state of local tissues in patients.

From a translational perspective, current evidence still derives mainly from animal experiments, *in vitro* studies, small exploratory clinical studies, and retrospective analyses, which remains insufficient to support metabolic intervention as a standard therapeutic strategy. Larger, prospective, multicenter clinical studies are therefore needed to systematically evaluate the safety, efficacy, optimal timing, and appropriate target populations of metabolic interventions in humans.

Given the marked heterogeneity of secondary lymphedema, clinical trials should stratify patients according to etiology, disease stage, and anatomical site, while also establishing standardized and reproducible systems for comprehensive therapeutic assessment. Beyond limb volume, future studies should incorporate additional outcome measures, including indocyanine green lymphography, lymphoscintigraphy, skin thickness, tissue stiffness, inflammatory cytokines, lipid mediator profiles, and oxidative stress markers.

In terms of therapeutic strategy, reliance on metabolic intervention alone or surgical reconstruction alone is unlikely to fully address the complex pathological basis of the disease. Future treatment paradigms should therefore place greater emphasis on the synergy between structural repair and local metabolic regulation. Accordingly, combining local metabolic therapy with lymphatic reconstructive surgery may represent an important direction for future lymphedema treatment.

## 11 Conclusions

The pathological progression of secondary lymphedema involves impaired lymphatic drainage, chronic inflammation, adipose deposition, and fibrosis, and is closely associated with persistent disruption of the local immunometabolic microenvironment. Long-term lymph stasis can induce multiple local metabolic abnormalities, including hypoxia, glycolytic reprogramming, mitochondrial dysfunction, oxidative stress, impaired fatty acid oxidation and ketone body oxidation, disrupted amino acid transport, increased saturated fatty acid burden, insulin resistance, lipid mediator imbalance, cholesterol retention, and adipokine dysregulation.

These metabolic alterations may further impair lymphatic endothelial-cell function and regulate immune-cell infiltration, macrophage polarization, and T-cell subset balance, thereby sustaining inflammation, adipose tissue remodeling, and fibrotic progression. Although metabolism-modulating agents show potential for improving the immunometabolic microenvironment in lymphedema, their therapeutic efficacy, safety, and suitable patient populations require further clinical validation.

## References

[B1] Aguirre V. Werner E. D. Giraud J. Lee Y. H. Shoelson S. E. White M. F. . (2002). Phosphorylation of Ser307 in insulin receptor substrate-1 blocks interactions with the insulin receptor and inhibits insulin action. J. Biol. Chem. 277, 1531–1537. doi: 10.1074/jbc.m101521200 11606564

[B2] Ahmed R. L. Thomas W. Yee D. Schmitz K. H . (2006). Randomized controlled trial of weight training and lymphedema in breast cancer survivors. J. Clin. Oncol. Off. J. Am. Soc. Clin. Oncol. 24, 2765–2772. doi: 10.1200/jco.2005.03.6749 16702582

[B3] Akl M. M. Ahmed A. (2025). Localized semaglutide injection for hyperinsulinemia-induced lymphatic dysfunction: A narrative review proposing a promising metabolic perspective for lymphedema therapy. Advanced Pharm. Bull. 15(3), 499–505. doi: 10.34172/apb.025.43911 41403736 PMC12703388

[B4] Aschen S. Zampell J. C. Elhadad S. Weitman E. De Brot M. Mehrara B. J. . (2012). Regulation of adipogenesis by lymphatic fluid stasis: Part II. Expression of adipose differentiation genes. Plast. Reconstructive Surg. 129, 838–847. doi: 10.1097/prs.0b013e3182450b47 22456356 PMC3445411

[B5] Aukland K. Reed R. K. (1993). Interstitial-lymphatic mechanisms in the control of extracellular fluid volume. Physiol. Rev. 73, 1–78. doi: 10.1152/physrev.1993.73.1.1 8419962

[B6] Baik J. E. Park H. J. Kataru R. P. Savetsky I. L. Ly C. L. Shin J. . (2022). TGF-β1 mediates pathologic changes of secondary lymphedema by promoting fibrosis and inflammation. Clin. Transl. Med. 12, e758. doi: 10.1002/ctm2.758 35652284 PMC9160979

[B7] Baluk P. Fuxe J. Hashizume H. Romano T. Lashnits E. Butz S. . (2007). Functionally specialized junctions between endothelial cells of lymphatic vessels. J. Exp. Med. 204, 2349–2362. doi: 10.1083/jcb1787oia15 17846148 PMC2118470

[B8] Baluk P. McDonald D. M. (2022). Buttons and zippers: endothelial junctions in lymphatic vessels. Cold Spring Harbor Perspect. Med. 12, a041178. doi: 10.1101/cshperspect.a041178 35534209 PMC9643678

[B9] Bertelli M. Kiani A. K. Paolacci S. Manara E. Dautaj A. Beccari T. . (2020). Molecular pathways involved in lymphedema: Hydroxytyrosol as a candidate natural compound for treating the effects of lymph accumulation. J. Biotechnol. 308, 82–86. doi: 10.1016/j.jbiotec.2019.11.017 31794783

[B10] Blum K. S. Karaman S. Proulx S. T. Ochsenbein A. M. Luciani P. Leroux J. C. . (2014). Chronic high-fat diet impairs collecting lymphatic vessel function in mice. PloS One 9, e94713. doi: 10.1371/journal.pone.0094713 24714646 PMC3979858

[B11] Brorson H. Ohlin K. Olsson G. Nilsson M . (2006). Adipose tissue dominates chronic arm lymphedema following breast cancer: an analysis using volume rendered CT images. Lymphatic Res. Biol. 4, 199–210. doi: 10.1089/lrb.2006.4404 17394403

[B12] Brown S. Tadros A. B. Montagna G. Bell T. Crowley F. Gallagher E. J. . (2024). Glucagon-like peptide-1 receptor agonists (GLP-1 RAs) may reduce the risk of developing cancer-related lymphedema following axillary lymph node dissection (ALND). Front. Pharmacol. 15, 1457363. doi: 10.3389/fphar.2024.1457363 39318780 PMC11420520

[B13] Bruns F. Büntzel J. Mücke R. Schönekaes K. Kisters K. Micke O. . (2004). Selenium in the treatment of head and neck lymphedema. Med. Principles Pract. 13, 185–190. doi: 10.1159/000078313 15181321

[B14] Chang T. C. Uen Y. H. Chou C. H. Sheu J. R. Chou D. S . (2013). The role of cyclooxygenase-derived oxidative stress in surgically induced lymphedema in a mouse tail model. Pharm. Biol. 51, 573–580. doi: 10.3109/13880209.2012.749923 23373707

[B15] Chen Z. Ghavimi S. A. A. Wu M. McNamara J. Barreiro O. Maridas D. . (2023). PPARγ agonist treatment reduces fibroadipose tissue in secondary lymphedema by exhausting fibroadipogenic PDGFRα+ mesenchymal cells. JCI Insight 8, e165324. doi: 10.1172/jci.insight.165324 38131378 PMC10807713

[B16] Cifarelli V. Appak-Baskoy S. Peche V. S. Kluzak A. Shew T. Narendran R. . (2021). Visceral obesity and insulin resistance associate with CD36 deletion in lymphatic endothelial cells. Nat. Commun. 12, 3350. doi: 10.1038/s41467-021-23808-3 34099721 PMC8184948

[B17] Cifarelli V. Eichmann A. (2019). The intestinal lymphatic system: functions and metabolic implications. Cell. Mol. Gastroenterol. Hepatol. 7, 503–513. doi: 10.1016/j.jcmgh.2018.12.002 30557701 PMC6396433

[B18] Clemente-Suárez V. J. Redondo-Flórez L. Beltrán-Velasco A. I. Martín-Rodríguez A. Martínez-Guardado I. Navarro-Jiménez E. . (2023). The role of adipokines in health and disease. Biomedicines 11, 1290. doi: 10.3390/biomedicines11051290 37238961 PMC10216288

[B19] Crowley F. Brown S. Gallagher E. J. Dayan J. H . (2024). GLP-1 receptor agonist as an effective treatment for breast cancer-related lymphedema: a case report. Front. Oncol. 14, 1392375. doi: 10.3389/fonc.2024.1392375 38699640 PMC11063291

[B20] Dang E. V. Barbi J. Yang H. Y. Jinasena D. Yu H. Zheng Y. . (2011). Control of TH17/Treg balance by hypoxia-inducible factor 1. Cell. 146, 772–784. doi: 10.1016/j.cell.2011.07.033 21871655 PMC3387678

[B21] Davis H. R. Compton D. S. Hoos L. Tetzloff G . (2001). Ezetimibe, a potent cholesterol absorption inhibitor, inhibits the development of atherosclerosis in ApoE knockout mice. Arteriosclerosis Thrombosis Vasc. Biol. 21, 2032–2038. doi: 10.1161/hq1201.100260 11742881

[B22] Davis M. J. Scallan J. P. Castorena-Gonzalez J. A. Kim H. J. Ying L. H. Pin Y. K. . (2023). Multiple aspects of lymphatic dysfunction in an ApoE-/- mouse model of hypercholesterolemia. Front. Physiol. 13, 1098408. doi: 10.3389/fphys.2022.1098408 36685213 PMC9852907

[B23] Dhuli K. Ceccarini M. R. Precone V. Maltese P. E. Bonetti G. Paolacci S. . (2021). Improvement of quality of life by intake of hydroxytyrosol in patients with lymphedema and association of lymphedema genes with obesity. 25(1), 33–42. doi: 10.26355/eurrev_202112_27331 34890032

[B24] Ding L. Zhang J. (2012). Glucagon-like peptide-1 activates endothelial nitric oxide synthase in human umbilical vein endothelial cells. Acta Pharmacol. Sin. 33, 75–81. doi: 10.1038/aps.2011.149 22120969 PMC4010269

[B25] Doruk Analan P. Kaya E. (2022). Is there a relationship between insulin resistance and breast cancer-related lymphedema? A preliminary study. Lymphatic Res. Biol. 20, 76–81. doi: 10.1089/lrb.2019.0072 33761281

[B26] Fujisaka S. Usui I. Ikutani M. Aminuddin A. Takikawa A. Tsuneyama K. . (2013). Adipose tissue hypoxia induces inflammatory M1 polarity of macrophages in an HIF-1α-dependent and HIF-1α-independent manner in obese mice. Diabetologia 56, 1403–1412. doi: 10.1007/s00125-013-2885-1 23494472

[B27] García-Caballero M. Zecchin A. Souffreau J. Truong A. C. K. Teuwen L. A. Vermaelen W. . (2019). Role and therapeutic potential of dietary ketone bodies in lymph vessel growth. Nat. Metab. 1, 666–675. doi: 10.1038/s42255-019-0087-y 32694649

[B28] García-Martín R. Alexaki V. I. Qin N. Rubín de Celis M. F. Economopoulou M. Ziogas A. . (2016). Adipocyte-specific hypoxia-inducible factor 2α deficiency exacerbates obesity-induced brown adipose tissue dysfunction and metabolic dysregulation. Mol. Cell. Biol. 36, 376–393. 26572826 10.1128/MCB.00430-15 PMC4719429

[B29] García-Martín R. Alexaki V. I. Qin N. Rubín de Celis M. F. Economopoulou M. Ziogas A. . (2016). Obesity but not high-fat diet impairs lymphatic function. Int. J. Obes. (2005) 40, 1582–1590. doi: 10.1128/MCB.00430-15 27200507 PMC5050064

[B30] García Nores G. D. Ly C. L. Cuzzone D. A. Kataru R. P. Hespe G. E. Torrisi J. S. . (2018). CD4+ T cells are activated in regional lymph nodes and migrate to skin to initiate lymphedema. Nat. Commun. 9, 1970. doi: 10.1038/s41467-018-04418-y 29773802 PMC5958132

[B31] Gardenier J. C. Hespe G. E. Kataru R. P. Savetsky I. L. Torrisi J. S. García Nores G. D. . (2016). Diphtheria toxin-mediated ablation of lymphatic endothelial cells results in progressive lymphedema. JCI Insight 1, e84095. doi: 10.1172/jci.insight.84095 27699240 PMC5033805

[B32] Ghanta S. Cuzzone D. A. Torrisi J. S. Albano N. J. Joseph W. J. Savetsky I. L. . (2015). Regulation of inflammation and fibrosis by macrophages in lymphedema. Am. J. Physiol. Heart Circ. Physiol. 308, H1065–H1077. doi: 10.1152/ajpheart.00598.2014 25724493 PMC4551121

[B33] Gomes K. P. Korodimas J. Liu E. Patel N. Yang X. Goruk S. . (2025). Saturated fatty acids induce lipotoxicity in lymphatic endothelial cells contributing to secondary lymphedema development. EMBO Mol. Med. 17, 2384–2408. doi: 10.1038/s44321-025-00286-4 40759794 PMC12423331

[B34] Gousopoulos E. Proulx S. T. Scholl J. Uecker M. Detmar M . (2016). Prominent lymphatic vessel hyperplasia with progressive dysfunction and distinct immune cell infiltration in lymphedema. Am. J. Pathol. 186, 2193–2203. doi: 10.1016/j.ajpath.2016.04.006 27315777

[B35] Guo X. Huang C. Zhang L. Hu G. Du Y. Chen X. . (2025). Lymphatic endothelial branched-chain amino acid catabolic defects undermine cardiac lymphatic integrity and drive HFpEF. Circulation 151, 1651–1666. doi: 10.1161/circulationaha.124.071741 40166847 PMC12144540

[B36] Han H. W. Yang E. J. Lee S. M. (2019). Sodium selenite alleviates breast cancer-related lymphedema independent of antioxidant defense system. Nutrients 11, 1021. doi: 10.3390/nu11051021 31067718 PMC6566195

[B37] Hartwig J. Loebel M. Steiner S. Bauer S. Karadeniz Z. Roeger C. . (2021). Metformin attenuates ROS via FOXO3 activation in immune cells. Front. Immunol. 12, 581799. doi: 10.3389/fimmu.2021.581799 33953705 PMC8089390

[B38] Hasenoehrl T. Palma S. Ramazanova D. Kölbl H. Dorner T. E. Keilani M. . (2020). Resistance exercise and breast cancer-related lymphedema-a systematic review update and meta-analysis. Supportive Care Cancer: Off. J. Multinational Assoc. Supportive Care Cancer 28, 3593–3603. doi: 10.1007/s00520-020-05521-x 32415386 PMC7316683

[B39] Hespe G. E. Kataru R. P. Savetsky I. L. García Nores G. D. Torrisi J. S. Nitti M. D. . (2016). Exercise training improves obesity-related lymphatic dysfunction. J. Physiol. 594, 4267–4282. doi: 10.1113/jp271757 26931178 PMC4967732

[B40] Hong Y. K. Harvey N. Noh Y. H. Schacht V. Hirakawa S. Detmar M. . (2002). Prox1 is a master control gene in the program specifying lymphatic endothelial cell fate. Dev. Dynamics: An. Off. Publ. Am. Assoc. Anatomists 225, 351–357. doi: 10.1002/dvdy.10163 12412020

[B41] Hossain L. Gomes K. P. Yang X. Liu E. Du Toit J. von der Weid P. Y. . (2024). Vascular endothelial growth factor C (VEGF-C) sensitizes lymphatic endothelial cells to oxidative-stress-induced apoptosis through DNA damage and mitochondrial dysfunction: Implications for lymphedema. Int. J. Mol. Sci. 25, 7828. doi: 10.3390/ijms25147828 39063073 PMC11277328

[B42] Hsiao H. Y. Liu J. W. Pappalardo M. Cheng M. H . (2023). The impacts of lymph on the adipogenesis of adipose-derived stem cells. Plast. Reconstructive Surg. 151, 1005–1015. doi: 10.1097/prs.0000000000010082 36534068

[B43] Hsu T. S. Lin Y. L. Wang Y. A. Mo S. T. Chi P. Y. Lai A. C. Y. . (2020). HIF-2α is indispensable for regulatory T cell function. Nat. Commun. 11, 5005. doi: 10.1038/s41467-020-18731-y 33024109 PMC7538433

[B44] Huang H. Vandekeere S. Kalucka J. Bierhansl L. Zecchin A. Brüning U. . (2017). Role of glutamine and interlinked asparagine metabolism in vessel formation. EMBO J. 36, 2334–2352. doi: 10.15252/embj.201695518 28659375 PMC5556263

[B45] Imtiyaz H. Z. Williams E. P. Hickey M. M. Patel S. A. Durham A. C. Yuan L. J. . (2010). Hypoxia-inducible factor 2α regulates macrophage function in mouse models of acute and tumor inflammation. J. Clin. Invest. 120, 2699–2714. doi: 10.1172/jci39506 20644254 PMC2912179

[B46] Ježek J. Cooper K. Strich R. (2018). Reactive oxygen species and mitochondrial dynamics: The yin and yang of mitochondrial dysfunction and cancer progression. Antioxidants 7, 13. doi: 10.3390/antiox7010013 29337889 PMC5789323

[B47] Jiang H. Zou Y. Zhao J. Li X. Yang S. Zhou X. . (2021). Pyruvate kinase M2 mediates glycolysis in the lymphatic endothelial cells and promotes the progression of lymphatic malformations. Am. J. Pathol. 191, 204–215. doi: 10.1016/j.ajpath.2020.10.003 33130045

[B48] Jiang X. Nicolls M. R. Tian W. Rockson S. G . (2018). Lymphatic dysfunction, leukotrienes, and lymphedema. Annu. Rev. Physiol. 80, 49–70. doi: 10.1146/annurev-physiol-022516-034008 29029593 PMC6434710

[B49] Jiang X. Tian W. Kim D. McQuiston A. S. Vinh R. Rockson S. G. . (2022). Hypoxia and hypoxia-inducible factors in lymphedema. Front. Pharmacol. 13, 851057. doi: 10.3389/fphar.2022.851057 35450048 PMC9017680

[B50] Jiang X. Tian W. Nicolls M. R. Rockson S. G . (2019). The lymphatic system in obesity, insulin resistance, and cardiovascular diseases. Front. Physiol. 10, 1402. doi: 10.3389/fphys.2019.01402 31798464 PMC6868002

[B51] Johandes E. Hall E. Harbut T. Priebe K. Schwarz M. A. Hanjaya-Putra D. . (2025). Blocking glutamine transport normalizes lymphatic vessels in hypoxic environments by attenuating glycolysis. doi: 10.64898/2025.12.18.695240

[B52] Karaman S. Lehti S. Zhang C. Taskinen M. Käkelä R. Mardinoglu A. . (2024). Multi‐omics characterization of lymphedema‐induced adipose tissue resulting from breast cancer‐related surgery. FASEB J. 38, e70097. doi: 10.1096/fj.202400498rr 39394863 PMC11580717

[B53] Karkkainen M. J. Haiko P. Sainio K. Partanen J. Taipale J. Petrova T. V. . (2004). Vascular endothelial growth factor C is required for sprouting of the first lymphatic vessels from embryonic veins. Nat. Immunol. 5, 74–80. doi: 10.1038/ni1013 14634646

[B54] Kasseroller R. G. Schrauzer G. N. (2000). Treatment of secondary lymphedema of the arm with physical decongestive therapy and sodium selenite: a review. Am. J. Ther. 7, 273–279. doi: 10.1097/00045391-200007040-00008 11486162

[B55] Kim D. J. Kim D. S. Yu Y. Chung J. H. Yoon E. S . (2024). Cross-sectional analysis for lymphedema epidemiology in South Korea by HIRA data: an observational study. Medicine 103, e38779. doi: 10.1097/md.0000000000038779 38968506 PMC11224808

[B56] Kondo Y. Jiang Y. Geng X. Song J. Simeroth S. McDaniel J. M. . (2025). Site-1 protease–mediated cholesterol metabolism is essential for lymphatic development in mice. 10(20), e188637. doi: 10.1172/jci.insight.188637 PMC12581667 41122969

[B57] Kukk E. Lymboussaki A. Taira S. Kaipainen A. Jeltsch M. Joukov V. . (1996). VEGF-C receptor binding and pattern of expression with VEGFR-3 suggests a role in lymphatic vascular development. Dev. (Cambridge England) 122, 3829–3837. doi: 10.1242/dev.122.12.3829 9012504

[B58] Lee P. Chandel N. S. Simon M. C. (2020). Cellular adaptation to hypoxia through hypoxia inducible factors and beyond. Nat. Rev. Mol. Cell Biol. 21, 268–283. doi: 10.1038/s41580-020-0227-y 32144406 PMC7222024

[B59] Lee S. Lee B. Kim Y. Min S. Yang E. Lee S. . (2021). Effects of sodium selenite injection on serum metabolic profiles in women diagnosed with breast cancer-related lymphedema—secondary analysis of a randomized placebo-controlled trial using global metabolomics. Nutrients 13, 3253. doi: 10.3390/nu13093253 34579131 PMC8470409

[B60] Levi B. Glotzbach J. P. Sorkin M. Hyun J. Januszyk M. Wan D. C. . (2013). Molecular analysis and differentiation capacity of adipose-derived stem cells from lymphedema tissue. Plast. Reconstructive Surg. 132, 580–589. doi: 10.1097/prs.0b013e31829ace13 23985633 PMC4447496

[B61] Liao S. Cheng G. Conner D. A. Huang Y. Kucherlapati R. S. Munn L. L. . (2011). Impaired lymphatic contraction associated with immunosuppression. PNAS 108, 18784–18789. doi: 10.1073/pnas.1116152108 22065738 PMC3219138

[B62] Lim H. Y. Zhang Y. Azhar S. H. M. Thiam C. H. Taylor M. Koh X. H. . (2026). Targeting excessive cholesterol deposition alleviates secondary lymphoedema. Nature 651, 462–471. doi: 10.1038/s41586-025-10016-y 41673147

[B63] Liu X. Yuan M. Xiang Q. Li Z. Xu F. Chen W. . (2022). Single-cell RNA sequencing of subcutaneous adipose tissues identifies therapeutic targets for cancer-associated lymphedema. Cell Discov. 8, 58. doi: 10.1038/s41421-022-00402-5 35725971 PMC9209506

[B64] Lodewijckx I. Matthys C. Verheijen J. Verscuren R. Devoogdt N. Van der Schueren B. . (2024). Potential therapeutic effect of a ketogenic diet for the treatment of lymphoedema: Results of an exploratory study. J. Hum. Nutr. Diet. 37, 885–891. doi: 10.1111/jhn.13330 38837503

[B65] Longo M. Zatterale F. Naderi J. Parrillo L. Formisano P. Raciti G. A. . (2019). Adipose tissue dysfunction as determinant of obesity-associated metabolic complications. Int. J. Mol. Sci. 20, 2358. doi: 10.3390/ijms20092358 31085992 PMC6539070

[B66] Lopez A. M. Terpack S. J. Posey K. S. Liu B. Ramirez C. M. Turley S. D. . (2014). Systemic administration of 2-hydroxypropyl-β-cyclodextrin to symptomatic Npc1-deficient mice slows cholesterol sequestration in the major organs and improves liver function. Clin. Exp. Pharmacol. Physiol. 41, 780–787. doi: 10.1111/1440-1681.12285 25115571 PMC4526211

[B67] Lübbering N. Krogmann A. Jansen F. Latz E. Nickenig G. Zimmer S. . (2025). Cyclodextrin reduces cholesterol crystal uptake by circulating monocytes in patients undergoing coronary angiography. PloS One 20, e0338635. doi: 10.1371/journal.pone.0338635 41396996 PMC12747169

[B68] Luo T. Nocon A. Fry J. Sherban A. Rui X. Jiang B. . (2016). AMPK activation by metformin suppresses abnormal extracellular matrix remodeling in adipose tissue and ameliorates insulin resistance in obesity. Diabetes 65, 2295–2310. doi: 10.2337/db15-1122 27207538 PMC4955985

[B69] Ma W. Gil H. J. Liu X. Diebold L. P. Morgan M. A. Oxendine-Burns M. J. . (2021). Mitochondrial respiration controls the Prox1-Vegfr3 feedback loop during lymphatic endothelial cell fate specification and maintenance. Sci. Adv. 7, eabe7359. doi: 10.1126/sciadv.abe7359 33931446 PMC8087398

[B70] Marques R. M. Gonzalez-Nunez M. Walker M. E. Gomez E. A. Colas R. A. Montero-Melendez T. . (2021). Loss of 15-lipoxygenase disrupts Treg differentiation altering their pro-resolving functions. Cell Death Differ. 28, 3140–3160. doi: 10.1038/s41418-021-00807-x 34040168 PMC8563763

[B71] Martin-Almedina S. Mortimer P. S. Ostergaard P. (2021). Development and physiological functions of the lymphatic system: insights from human genetic studies of primary lymphedema. Physiol. Rev. 101, 1809–1871. doi: 10.1152/physrev.00006.2020 33507128

[B72] Mazzatti D. Lim F. L. O'Hara A. Wood I. S. Trayhurn P . (2012). A microarray analysis of the hypoxia-induced modulation of gene expression in human adipocytes. Arch. Physiol. Biochem. 118, 112–120. doi: 10.3109/13813455.2012.654611 22352407

[B73] Mehrara B. (2026). Clearing trapped cholesterol could relieve lymphoedema. Nature 651, 314–315. doi: 10.1038/d41586-026-00176-w 41673477

[B74] Micke O. Bruns F. Mücke R. Schäfer U. Glatzel M. DeVries A. F. . (2003). Selenium in the treatment of radiation-associated secondary lymphedema. Int. J. Radiat. Oncol. Biol. Phys. 56, 40–49. doi: 10.1016/s0360-3016(02)04390-0 12694822

[B75] Min Y. Ghose S. Boelte K. Li J. Yang L. Lin P. C. . (2011). C/EBP-δ regulates VEGF-C autocrine signaling in lymphangiogenesis and metastasis of lung cancer through HIF-1α. Oncogene 30, 4901–4909. doi: 10.1038/onc.2011.187 21666710 PMC3175299

[B76] Morris C. J. Kameny R. J. Boehme J. Gong W. He Y. Zhu T. . (2018). KLF2-mediated disruption of PPAR-γ signaling in lymphatic endothelial cells exposed to chronically increased pulmonary lymph flow. Am. J. Physiol. Heart Circ. Physiol. 315, H173–H181. doi: 10.1152/ajpheart.00635.2017 29631374 PMC6087778

[B77] Mraz M. Haluzik M. (2014). The role of adipose tissue immune cells in obesity and low-grade inflammation. J. Endocrinol. 222, R113–R127. doi: 10.1530/joe-14-0283 25006217

[B78] Murdaca G. Cagnati P. Gulli R. Spanò F. Puppo F. Campisi C. . (2012). Current views on diagnostic approach and treatment of lymphedema. Am. J. Med. 125, 134–140. doi: 10.1016/j.amjmed.2011.06.032 22269614

[B79] Murtomaki A. Uh M. K. Kitajewski C. Zhao J. Nagasaki T. Shawber C. J. . (2014). Notch signaling functions in lymphatic valve formation. Development 141, 2446–2456. doi: 10.1242/dev.101188 24917500 PMC4050693

[B80] Nakamura K. Radhakrishnan K. Wong Y. M. Rockson S. G . (2009). Anti-inflammatory pharmacotherapy with ketoprofen ameliorates experimental lymphatic vascular insufficiency in mice. PloS One 4, e8380. doi: 10.1371/journal.pone.0008380 20027220 PMC2791214

[B81] Nguyen M. T. A. Favelyukis S. Nguyen A. K. Reichart D. Scott P. A. Jenn A. . (2007). A subpopulation of macrophages infiltrates hypertrophic adipose tissue and is activated by free fatty acids via Toll-like receptors 2 and 4 and JNK-dependent pathways. J. Biol. Chem. 282, 35279–35292. doi: 10.1074/jbc.m706762200 17916553

[B82] Norden P. R. Kume T. (2020). The role of lymphatic vascular function in metabolic disorders. Front. Physiol. 11, 404. doi: 10.3389/fphys.2020.00404 32477160 PMC7232548

[B83] Ogata F. Fujiu K. Matsumoto S. Nakayama Y. Shibata M. Oike Y. . (2016). Excess lymphangiogenesis cooperatively induced by macrophages and CD4+ T cells drives the pathogenesis of lymphedema. J. Invest. Dermatol. 136, 706–714. doi: 10.1016/j.jid.2015.12.001 27015456

[B84] Ohhashi T. Kawai Y. Maejima D. Hayashi M. Watanabe-Asaka T . (2023). Physiological roles of lymph flow-mediated nitric oxide in lymphatic system. Lymphatic Res. Biol. 21, 253–261. doi: 10.1089/lrb.2022.0072 36577034

[B85] Oliver G. Kipnis J. Randolph G. J. Harvey N. L . (2020). The lymphatic vasculature in the 21(st) century: novel functional roles in homeostasis and disease. Cell. 182, 270–296. doi: 10.1016/j.cell.2020.06.039 32707093 PMC7392116

[B86] Oliver G. Srinivasan R. S. (2010). Endothelial cell plasticity: how to become and remain a lymphatic endothelial cell. Dev. (Cambridge England) 137, 363–372. doi: 10.1242/dev.035360 20081185 PMC2858906

[B87] Panchapakesan U. Pollock C. Saad S. (2009). Review article: Importance of the kidney proximal tubular cells in thiazolidinedione-mediated sodium and water uptake. Nephrol. (Carlton Vic.) 14, 298–301. doi: 10.1111/j.1440-1797.2009.01089.x 19444964

[B88] Park H. J. Shin J. Sarker A. Klang M. G. Riedel E. Coriddi M. . (2025). TGF-β-mediated epithelial-mesenchymal transition of keratinocytes promotes fibrosis in secondary lymphedema. JCI Insight 10, e192890. doi: 10.1172/jci.insight.192890 40728885 PMC12487678

[B89] Pfister C. Dawzcynski H. Schingale F. J. (2016). Sodium selenite and cancer related lymphedema: Biological and pharmacological effects. J. Trace Elem. Med. Biol. 37, 111–116. doi: 10.1016/j.jtemb.2016.05.005 27267968

[B90] Puchalska P. Crawford P. A. (2017). Multi-dimensional roles of ketone bodies in fuel metabolism, signaling, and therapeutics. Cell Metab. 25, 262–284. doi: 10.1016/j.cmet.2016.12.022 28178565 PMC5313038

[B91] Randolph G. J. Ivanov S. Zinselmeyer B. H. Scallan J. P . (2017). The lymphatic system: integral roles in immunity. Annu. Rev. Immunol. 35, 31–52. doi: 10.1146/annurev-immunol-041015-055354 27860528 PMC5551392

[B92] Randolph G. J. Miller N. E. (2014). Lymphatic transport of high-density lipoproteins and chylomicrons. J. Clin. Invest. 124, 929–935. doi: 10.18572/2658-7130-2025-1-2-22-34 24590278 PMC3934183

[B93] Rockson S. G. Tian W. Jiang X. Kuznetsova T. Haddad F. Zampell J. . (2018). Pilot studies demonstrate the potential benefits of antiinflammatory therapy in human lymphedema. JCI Insight 3, e123775. doi: 10.1172/jci.insight.123775 30333315 PMC6237444

[B94] Rubin J. Pollack B. Coleman-Belin J. Campbell A. C. Roberts A. Wagner B. D. . (2025). Metformin use and risk of breast cancer-related lymphedema: A retrospective analysis. J. Am. Coll. Surgeons 241, 637–646. doi: 10.1097/xcs.0000000000001434 40323038 PMC12419834

[B95] Sanz A. Caro P. Gómez J. Barja G . (2006). Testing the vicious cycle theory of mitochondrial ROS production: Effects of H2O2 and cumene hydroperoxide treatment on heart mitochondria. J. Bioenerg. Biomembr. 38, 121–127. doi: 10.1007/s10863-006-9011-8 16841200

[B96] Sato A. Kamekura R. Kawata K. Kawada M. Jitsukawa S. Yamashita K. . (2016). Novel mechanisms of compromised lymphatic endothelial cell homeostasis in obesity: The role of leptin in lymphatic endothelial cell tube formation and proliferation. PloS One 11, e0158408. doi: 10.1371/journal.pone.0158408 27366905 PMC4930203

[B97] Schmitz K. H. Ahmed R. L. Troxel A. Cheville A. Smith R. Lewis-Grant L. . (2009). Weight lifting in women with breast-cancer-related lymphedema. N. Engl. J. Med. 361, 664–673. doi: 10.1056/nejmoa0810118 19675330

[B98] Schoors S. Bruning U. Missiaen R. Queiroz K. C. S. Borgers G. Elia I. . (2015). Fatty acid carbon is essential for dNTP synthesis in endothelial cells. Nature 520, 192–197. doi: 10.1038/nature14362 25830893 PMC4413024

[B99] Schulz M. E. Akerstrom V. L. Dayan J. H. Castorena-Gonzalez J. A . (2025). GLP-1R agonism directly improves the pumping capacity of murine collecting lymphatic vessels. doi: 10.64898/2025.12.26.696603 PMC13436613 42231627

[B100] Schwager S. Detmar M. (2019). Inflammation and lymphatic function. Front. Immunol. 10, 308. doi: 10.3389/fimmu.2019.00308 30863410 PMC6399417

[B101] Shaw C. Mortimer P. Judd P. A. (2007a). A randomized controlled trial of weight reduction as a treatment for breast cancer‐related lymphedema. Cancer 110, 1868–1874. doi: 10.1002/cncr.22638 17823909

[B102] Shaw C. Mortimer P. Judd P. A. (2007b). Randomized controlled trial comparing a low‐fat diet with a weight‐reduction diet in breast cancer‐related lymphedema. Cancer 109, 1949–1956. 17393377 10.1002/cncr.22638

[B103] Shi H. Kokoeva M. V. Inouye K. Tzameli I. Yin H. Flier J. S. . (2006). TLR4 links innate immunity and fatty acid-induced insulin resistance. J. Clin. Invest. 116, 3015–3025. doi: 10.1172/jci28898 17053832 PMC1616196

[B104] Shimizu Y. Shibata R. Ishii M. Ohashi K. Kambara T. Uemura Y. . (2013). Adiponectin‐mediated modulation of lymphatic vessel formation and lymphedema. J. Am. Heart Assoc. 2, e000438. doi: 10.1161/jaha.113.000438 24052499 PMC3835259

[B105] Shirouchi B. Nakamura Y. Furukawa Y. Shiraishi A. Tomoyori H. Imaizumi K. . (2012). Ezetimibe inhibits lymphatic transport of esterified cholesterol but not free cholesterol in thoracic lymph duct-cannulated rats. Cardiovasc. Drugs Ther. 26, 427–431. doi: 10.1007/s10557-012-6403-3 22798197

[B106] Siems W. G. (2002). Oxidative stress in chronic lymphoedema. QJM 95, 803–809. doi: 10.1093/qjmed/95.12.803 12454323

[B107] Simeroth S. Yu P. (2024). The role of lymphatic endothelial cell metabolism in lymphangiogenesis and disease. Front. Cardiovasc. Med. 11, 1392816. doi: 10.3389/fcvm.2024.1392816 38798921 PMC11119333

[B108] Singh N. K. Rao G. N. (2019). Emerging role of 12/15-lipoxygenase (ALOX15) in human pathologies. Prog. Lipid Res. 73, 28–45. doi: 10.1016/j.plipres.2018.11.001 30472260 PMC6338518

[B109] Srinivasan R. S. Escobedo N. Yang Y. Interiano A. Dillard M. E. Finkelstein D. . (2014). The Prox1-Vegfr3 feedback loop maintains the identity and the number of lymphatic endothelial cell progenitors. Genes Dev. 28, 2175–2187. doi: 10.1101/gad.216226.113 25274728 PMC4180978

[B110] Srinivasan R. S. Geng X. Yang Y. Wang Y. Mukatira S. Studer M. . (2010). The nuclear hormone receptor Coup-TFII is required for the initiation and early maintenance of Prox1 expression in lymphatic endothelial cells. Genes Dev. 24, 696–707. doi: 10.1101/gad.1859310 20360386 PMC2849126

[B111] Sudduth C. L. Greene A. K. (2022). Lymphedema and obesity. Cold Spring Harbor Perspect. Med. 12, a041176. doi: 10.1101/cshperspect.a041176 35074795 PMC9159261

[B112] Taguchi K. Fukami K. (2023). RAGE signaling regulates the progression of diabetic complications. Front. Pharmacol. 14, 1128872. doi: 10.3389/fphar.2023.1128872 37007029 PMC10060566

[B113] Tashiro K. Feng J. Wu S. H. Mashiko T. Kanayama K. Narushima M. . (2017). Pathological changes of adipose tissue in secondary lymphoedema. Br. J. Dermatol. 177, 158–167. doi: 10.1111/bjd.15238 28000916

[B114] Tian W. Rockson S. G. Jiang X. Kim J. Begaye A. Shuffle E. M. . (2017). Leukotriene B_4_ antagonism ameliorates experimental lymphedema. Sci. Transl. Med. 9, eaal3920. doi: 10.1007/978-981-16-0784-4_41 28490670

[B115] Toki S. Newcomb D. C. Printz R. L. Cahill K. N. Boyd K. L. Niswender K. D. . (2021). Glucagon‐like peptide‐1 receptor agonist inhibits aeroallergen‐induced activation of ILC2 and neutrophilic airway inflammation in obese mice. Allergy 76, 3433–3445. doi: 10.1111/all.14879 33955007 PMC8597133

[B116] Trasca D. M. Mocanu A. G. Pluta I. D. Popescu C. Stoica G. A. Varut R. M. . (2026). Cyclodextrins as modulators of regulated cell death: Implications for immunometabolism and therapeutic innovation. Pharmaceutics 18, 306. doi: 10.3390/pharmaceutics18030306 41900791 PMC13029772

[B117] Turrens J. F. (1997). Superoxide production by the mitochondrial respiratory chain. Biosci. Rep. 17, 3–8. doi: 10.1023/a:1027374931887 9171915

[B118] Vafa S. Zarrati M. Malakootinejad M. Totmaj A. S. Zayeri F. Salehi M. . (2020). Calorie restriction and synbiotics effect on quality of life and edema reduction in breast cancer-related lymphedema, a clinical trial. Breast (Edinburgh Scotland) 54, 37–45. doi: 10.1016/j.breast.2020.08.008 32898787 PMC7486474

[B119] Wei X. L. Tao M. H. Li R. H. Ge S. H. Xiao W . (2025). Metformin and adipose tissue: A multifaceted regulator in metabolism, inflammation, and regeneration. Endocrinol. Metab. 40, 523–538. doi: 10.3803/enm.2025.2371 40775736 PMC12409170

[B120] Wei M. Wang L. Liu X. Deng Y. Yang S. Pan W. . (2024). Metformin eliminates lymphedema in mice by alleviating inflammation and fibrosis: Implications for human therapy. Plast. Reconstructive Surg. 154, 1128e–1137e. doi: 10.1097/prs.0000000000011363 38391208 PMC11584190

[B121] Weis E. Puchalska P. Nelson A. B. Taylor J. Moll I. Hasan S. S. . (2022). Ketone body oxidation increases cardiac endothelial cell proliferation. EMBO Mol. Med. 14, e14753. doi: 10.15252/emmm.202114753 35179309 PMC8988203

[B122] Weitman E. S. Aschen S. Z. Farias-Eisner G. Albano N. Cuzzone D. A. Ghanta S. . (2013). Obesity impairs lymphatic fluid transport and dendritic cell migration to lymph nodes. PloS One 8, e70703. doi: 10.1371/journal.pone.0070703 23950984 PMC3741281

[B123] Wigle J. T. Harvey N. Detmar M. Lagutina I. Grosveld G. Gunn M. D. . (2002). An essential role for Prox1 in the induction of the lymphatic endothelial cell phenotype. EMBO J. 21, 1505–1513. doi: 10.1093/emboj/21.7.1505 11927535 PMC125938

[B124] Wigle J. T. Oliver G. (1999). Prox1 function is required for the development of the murine lymphatic system. Cell. 98, 769–778. doi: 10.1016/s0092-8674(00)81511-1 10499794

[B125] Wilting J. Becker J. (2022). The lymphatic vascular system: much more than just a sewer. Cell. Bioscience 12, 157. doi: 10.1186/s13578-022-00898-0 36109802 PMC9476376

[B126] Wong B. W. Wang X. Zecchin A. Thienpont B. Cornelissen I. Kalucka J. . (2017). The role of fatty acid β-oxidation in lymphangiogenesis. Nature 542, 49–54. doi: 10.1038/nature21028 28024299

[B127] Wong B. W. Zecchin A. García-Caballero M. Carmeliet P . (2018). Emerging concepts in organ-specific lymphatic vessels and metabolic regulation of lymphatic development. Dev. Cell 45, 289–301. doi: 10.1016/j.devcel.2018.03.021 29738709

[B128] Wueest S. Rapold R. A. Rytka J. M. Schoenle E. J. Konrad D . (2009). Basal lipolysis, not the degree of insulin resistance, differentiates large from small isolated adipocytes in high-fat fed mice. Diabetologia 52, 541–546. doi: 10.1007/s00125-008-1223-5 19048227

[B129] Xiang Q. Xu F. Li Y. Liu X. Chen Q. Huang J. . (2020). Transcriptome analysis and functional identification of adipose-derived mesenchymal stem cells in secondary lymphedema. Gland Surg. 9, 558–574. doi: 10.21037/gs.2020.02.09 32420291 PMC7225476

[B130] Xie N. Tan Z. Banerjee S. Cui H. Ge J. Liu R. M. . (2015). Glycolytic reprogramming in myofibroblast differentiation and lung fibrosis. Am. J. Respir. Crit. Care Med. 192, 1462–1474. doi: 10.1164/rccm.201504-0780oc 26284610 PMC4731722

[B131] Yang Y. García-Verdugo J. M. Soriano-Navarro M. Srinivasan R. S. Scallan J. P. Singh M. K. . (2012). Lymphatic endothelial progenitors bud from the cardinal vein and intersomitic vessels in mammalian embryos. Blood 120, 2340–2348. doi: 10.1182/blood-2012-05-428607 22859612 PMC3447786

[B132] Yang J. C. S. Huang L. H. Wu S. C. Kuo P. J. Wu Y. C. Wu C. J. . (2021). Lymphaticovenous anastomosis supermicrosurgery decreases oxidative stress and increases antioxidant capacity in the serum of lymphedema patients. J. Clin. Med. 10, 1540. doi: 10.3390/jcm10071540 33917571 PMC8038828

[B133] Yang S. P. Su Q. Zhang Y. R. Sun Y. Chai Y. R . (2022). Metformin ameliorates thymus degeneration of mice by regulating mitochondrial function. Int. Immunopharmacol. 108, 108744. doi: 10.1016/j.intimp.2022.108744 35395467

[B134] Ye J. M. Dzamko N. Cleasby M. E. Hegarty B. D. Furler S. M. Cooney J. Y. (2004). Direct demonstration of lipid sequestration as a mechanism by which rosiglitazone prevents fatty-acid-induced insulin resistance in the rat: comparison with metformin. Diabetologia 47, 1306–1313. doi: 10.1007/s00125-004-1436-1 15232684

[B135] Yin J. Gao Z. He Q. Zhou D. Guo Z. Ye J. (2009). Role of hypoxia in obesity-induced disorders of glucose and lipid metabolism in adipose tissue. Am. J. Physiol. Endocrinol. Metab. 296, E333–E342. doi: 10.1152/ajpendo.90760.2008 19066318 PMC2645021

[B136] Yu P. Alves T. C. Kibbey R. G. . (2018). Metabolic analysis of lymphatic endothelial cells. Methods Mol. Biol. (Clifton N.J.) 1846, 325–334. doi: 10.1007/978-1-4939-8712-2_22 30242770 PMC6693514

[B137] Yu P. Wilhelm K. Dubrac A. Tung J. K. Alves T. C. Fang J. S. (2017). FGF-dependent metabolic control of vascular development. Nature 545, 224–228. doi: 10.1016/j.jvs.2017.07.055 28467822 PMC5427179

[B138] Yusof K. M. Groen K. Rosli R. Abdullah M. Mahmud R. Avery-Kiejda K. A. . (2022). Evaluation of circulating microRNAs and adipokines in breast cancer survivors with arm lymphedema. Int. J. Mol. Sci. 23, 11359. doi: 10.3390/ijms231911359 36232660 PMC9570352

[B139] Zaleska M. T. Olszewski W. L. (2017). Serum immune proteins in limb lymphedema reflecting tissue processes caused by lymph stasis and chronic dermato-lymphangio-adenitis (cellulitis). Lymphatic Res. Biol. 15, 246–251. doi: 10.1089/lrb.2017.0003 28880710

[B140] Zamora A. Nougué M. Verdu L. Balzan E. Draia-Nicolau T. Benuzzi E. . (2024). 15-lipoxygenase promotes resolution of inflammation in lymphedema by controlling Treg cell function through IFN-β. Nat. Commun. 15, 221. doi: 10.1038/s41467-023-43554-y 38177096 PMC10766617

[B141] Zampell J. C. Yan A. Avraham T. Daluvoy S. Weitman E. S. Mehrara B. J. . (2012a). HIF-1α coordinates lymphangiogenesis during wound healing and in response to inflammation. FASEB Journal: Off. Publ. Fed. Am. Societies For. Exp. Biol. 26, 1027–1039. doi: 10.1096/fj.11-195321 22067482 PMC3470728

[B142] Zampell J. C. Yan A. Elhadad S. Avraham T. Weitman E. Mehrara B. J. . (2012b). CD4+ cells regulate fibrosis and lymphangiogenesis in response to lymphatic fluid stasis. PloS One 7, e49940. doi: 10.1371/journal.pone.0049940 23185491 PMC3502174

[B143] Zhang J. Hoffner M. Brorson H. (2022). Adipocytes are larger in lymphedematous extremities than in controls. J. Plast. Surg. Handb. Surg. 56, 172–179. doi: 10.1080/2000656x.2021.1953042 34339353

[B144] Zhang D. Tang Z. Huang H. Zhou G. Cui C. Weng Y. . (2019). Metabolic regulation of gene expression by histone lactylation. Nature 574, 575–580. doi: 10.1038/s41586-019-1678-1 31645732 PMC6818755

[B145] Zimmermann T. Leonhardt H. Kersting S. Albrecht S. Range U. Eckelt U. . (2005). Reduction of postoperative lymphedema after oral tumor surgery with sodium selenite. Biol. Trace Elem. Res. 106, 193–204. doi: 10.1385/bter:106:3:193 16141467

